# BDNF-TrkB Signaling Coupled to nPKCε and cPKCβI Modulate the Phosphorylation of the Exocytotic Protein Munc18-1 During Synaptic Activity at the Neuromuscular Junction

**DOI:** 10.3389/fnmol.2018.00207

**Published:** 2018-06-12

**Authors:** Anna Simó, Laia Just-Borràs, Víctor Cilleros-Mañé, Erica Hurtado, Laura Nadal, Marta Tomàs, Neus Garcia, Maria A. Lanuza, Josep Tomàs

**Affiliations:** Unitat d'Histologia i Neurobiologia, Facultat de Medicina i Ciències de la Salut, Universitat Rovira i Virgili, Reus, Spain

**Keywords:** neuromuscular junction, muscle contraction, Munc18-1, BDNF-TrkB pathway, neurotrophic factors, PKC isoforms, neurotransmission, synaptic vesicles

## Abstract

Munc18-1, a neuron-specific member of the Sec1/Munc18 family, is involved in neurotransmitter release by binding tightly to syntaxin. Munc18-1 is phosphorylated by PKC on Ser-306 and Ser-313 *in vitro* which reduces the amount of Munc18-1 able to bind syntaxin. We have previously identified that PKC is involved in neurotransmitter release when continuous electrical stimulation imposes a moderate activity on the NMJ and that muscle contraction through TrkB has an important impact on presynaptic PKC isoforms levels, specifically cPKCβI and nPKCε. Therefore, the present study was designed to understand how Munc18-1 phosphorylation is affected by (1) synaptic activity at the neuromuscular junction, (2) nPKCε and cPKCβI isoforms activity, (3) muscle contraction *per se*, and (4) the BDNF/TrkB signaling in a neuromuscular activity-dependent manner. We performed immunohistochemistry and confocal techniques to evidence the presynaptic location of Munc18-1 in the rat diaphragm muscle. To study synaptic activity, we stimulated the phrenic nerve (1 Hz, 30 min) with or without contraction (abolished by μ-conotoxin GIIIB). Specific inhibitory reagents were used to block nPKCε and cPKCβI activity and to modulate the tropomyosin receptor kinase B (TrkB). Main results obtained from Western blot experiments showed that phosphorylation of Munc18-1 at Ser-313 increases in response to a signaling mechanism initiated by synaptic activity and directly mediated by nPKCε. Otherwise, cPKCβI and TrkB activities work together to prevent this synaptic activity–induced Munc18-1 phosphorylation by a negative regulation of cPKCβI over nPKCε. Therefore, a balance between the activities of these PKC isoforms could be a relevant cue in the regulation of the exocytotic apparatus. The results also demonstrate that muscle contraction prevents the synaptic activity–induced Munc18-1 phosphorylation through a mechanism that opposes the TrkB/cPKCβI/nPKCε signaling.

## Introduction

Synapse functionality is the result of several signaling pathways converging on intracellular kinases, which phosphorylate protein targets to perform adequate adaptive changes (Tomàs et al., 2014). During spontaneous and activity-evoked ACh release, presynaptic receptors and their coupled serine-threonine kinases A (PKA) and C (PKC) act simultaneously to adjust neurotransmission through the voltage-dependent calcium channels (VDCC) and the ready releasable pool of synaptic vesicles which are the instruments of transmitter release (Takamori, 2012).

Munc18-1 is an essential, neuron-specific protein from the SEC1 family involved in neurotransmitter release. Its knockouts show complete loss of neurotransmission and die immediately after birth (Verhage et al., 2000). Its functionality is orchestrated by its two PKC phosphorylation sites Ser 306 and Ser-313 (Fujita et al., 1996; Barclay et al., 2003; Morgan et al., 2004; Snyder et al., 2006). One of its roles is priming vesicle fusion and increasing the pool available for release (Sons et al., 2003; Südhof, 2013). Moreover, Munc18-1 tightly binds to syntaxin and holds it in a closed conformation to prevent SNARE assembly together with synaptobrevin and SNAP-25 (Hata et al., 1993; Dulubova et al., 1999; Misura et al., 2000; Yang et al., 2000; Liu et al., 2004; Khvotchev et al., 2007; Medine et al., 2007; Shen et al., 2007; Südhof and Rothman, 2009; Smyth et al., 2010a,b). Also, it works as a chaperone by delivering syntaxin to the plasma membrane (Arunachalam et al., 2007; Han et al., 2009) and, when associated with the SNARE complex, favors lipid mixing between membranes (Huang et al., 2011; Südhof, 2013).

In the neuromuscular system, PKC signaling is fundamental for the NMJ function. Although Munc18-1 is a PKC target in neurons, no information is known about which isoform is involved in the regulation of its phosphorylation in the NMJ. There are several PKC isoforms involved in neurotransmitter release (Vaughan et al., 1998; Hilfiker and Augustine, 1999; Leenders and Sheng, 2005; Santafé et al., 2005, 2006; Korogod et al., 2007; Genc et al., 2014; Katayama et al., 2017), which are differentially distributed at the NMJ (Hilgenberg and Miles, 1995; Lanuza et al., 2000; Perkins et al., 2001; Li et al., 2004; Besalduch et al., 2010, 2013; Obis et al., 2015a). Due to their nerve terminal distribution and involvement in acetylcholine release, cPKCβI and nPKCε are good candidates to regulate Munc18-1 phosphorylation (Besalduch et al., 2010; Obis et al., 2015a,b; Hurtado et al., 2017b). It has been shown that nerve-induced muscle contraction retrogradely enhances nerve terminal function by increasing brain-derived neurotrophic factor (BDNF) levels and decreasing the negative action of truncated isoform-tropomyosin receptor kinase B (TrkB.T1) over the full-length isoform (TrkB.FL) (Hurtado et al., 2017b). Presynaptic activity couples PKC to neurotransmitter release and muscle contraction restores presynaptic PKC isoforms βI and ε through TrkB signaling (Obis et al., 2015b; Hurtado et al., 2017b). Here, we analyze how Munc18-1 is regulated by synaptic activity and how BDNF/TrkB signaling through specific PKC isoforms could regulate its phosphorylation in the NMJ. Results show that Munc18-1 phosphorylation at the NMJ is increased in response to a signaling mechanism initiated by synaptic activity and directly mediated by nPKCε while cPKCβI and TrkB activities work to prevent it. Muscle contraction also prevents Munc18-1 phosphorylation and nPKCε, cPKCβI, and TrkB activities are involved suggesting that postsynaptic contraction could regulate presynaptic nPKCε and cPKCβI activities through TrkB.

## Materials and methods

### Animals

Young adult Sprague-Dawley rats (30–40 days; Criffa, Barcelona, Spain; RRID:RGD_5508397) were cared for in accordance with the guidelines of the European Community Council Directive for the humane treatment of laboratory animals. All the procedures realized were reviewed and approved by the Animal Research Committee of the Universitat Rovira i Virgili. Diaphragm, *levator auris longus* (LAL) muscle, brain and spinal cord were quickly removed and transferred to ice-cold dissection Ringer. At least three animals were used to evaluate the following techniques.

### Antibodies

Primary antibodies purchased from Santa Cruz Biotechnology: rabbit anti-PKCβI (Cat# sc-209 RRID:AB_2168968), rabbit anti-PKCε (Cat# sc-214 RRID:AB_2237729), goat anti-pPKCε (Ser 729) (Cat# sc-12355 RRID:AB_2171921) polyclonal antibodies and mouse monoclonal anti-GAPDH (Cat# sc-32233 RRID:AB_627679). Primary antibodies purchased from Abcam: Rabbit anti-pPKCβI (Thr 642) (Cat# RRID:AB_1310586), anti-pMunc18-1 (Ser-313) (Cat# ab138687) polyclonal antibodies. Rabbit monoclonal Munc18-1 (D4O6V) antibody was purchased from Cell Signaling Technologies (Cat# 13414) and mouse monoclonal Na^+^/K^+^-ATPase from Developmental Studies Hybridoma Bank (Cat# RRID:AB_528092).

The secondary antibodies conjugated to HRP were donkey anti-rabbit from Jackson Immunoresearch Labs (Cat# 711-035-152 RRID:AB_10015282), Rabbit anti-mouse from Sigma (Cat# A9044 RRID:AB_258431) and rabbit anti-goat from Molecular probes (Cat# R21459 RRID:AB_11180332). Immunohistochemistry was performed with antibodies widely utilized as markers of NMJ neuron and Schwann cell (syntaxin, neurofilament-200, and S-100): mouse anti-syntaxin (Cat# S0664 RRID:AB_477483) and mouse anti-neurofilament-200 (Cat# N2912 RRID:AB_477262) monoclonal antibodies from Sigma. Mouse anti-S-100 monoclonal antibody (Cat# AM10036FC-N RRID:AB_1622661) from Acris, Germany. The secondary antibodies used were donkey anti-rabbit or anti-mouse conjugated to Alexa Fluor 488 and Alexa Fluor 647 from Molecular Probes (Eugene, OR) (Cat# A21206 RRID:AB_141708; Cat# A21202 RRID:AB_141607; Cat# A-31573 RRID:AB_2536183; Cat# A-31571 RRID:AB_2536181). Postsynaptic AChRs were detected with α-bungarotoxin (α-BTX) conjugated to TRITC from Molecular Probes (Eugene, OR) (Cat# T1175 RRID:AB_2313931).

Primary antibodies were omitted in some immunohistochemical and Western blot procedures as negative control. These controls never exhibited positive staining or HRP activity with the respective procedures. In double-staining protocols, the omission of either one of the two primary antibodies completely abolished the corresponding staining with no cross-reaction with the other primary antibody. Antibody specificity against PKC isoforms is shown in Obis et al. (2015a) and Hurtado et al. (2017b). The anti-Munc18-1 antibody was raised against Y157 and its surrounding residues, which are not conserved in other Munc18 isoforms and the anti-pMunc18-1 Ser-313 antibody was raised against a synthetic peptide corresponding to the human Munc18-1 residues around the PKC target (307–319).

### Reagents

Muscle contraction was blocked using μ-conotoxin GIIIB (μ-CgTx-GIIIB, Alomone Labs Ltd, Jerusalem, Israel) for the presynaptic stimulation treatment (see below). This peptide selectively inhibits sarcolemmal voltage-dependent sodium channels (VDSCs) without altering ACh signaling (Favreau et al., 1999). It was supplied as lyophilised powder of >99% purity. The working concentration was 1.5 μM in Ringer's solution (see below).

TrkB inhibition assays were performed using an anti-TrkB antibody (clone 47/TrkB) obtained from BD Transduction Laboratories (Cat# 610101 RRID:AB_397507). This antibody has been functionally validated as a TrkB selective inhibitor, reducing BDNF effects without binding to TrkA, TrkC nor p75^NTR^ (Cazorla et al., 2011). The working solution of 47/TrkB was 10 μg/ml. For BDNF exogenous incubations we used h-BDNF (Alomone Labs; Cat# B-250) in a working solution of 10 mM.

Phosphatase inhibition experiments were performed using a phosphatase inhibitor cocktail obtained from Sigma-Aldrich Corporation (Saint Louis, MO, USA) in a 100-fold dilution.

PKC activation experiments were performed applying high calcium (Ca^2+^) concentration (5 mM of Ca^2+^) and phorbol 12-myristate 13-acetate (PMA) (10 nM; Santafé et al., 2005) in muscle and 100 nM in brain (Wierda et al., 2007). PKC inhibition experiments were performed applying low Ca^2+^ concentration (0.25 mM of Ca^2+^) and specific translocation inhibitors. The specific cPKCβI inhibitor βIV_5−3_ peptide (Liu et al., 1999; Zhang et al., 2015) was kindly provided by Dr. Mochly-Rosen from Stanford University and the specific translocation inhibitor nPKCε (εV_1−2_ peptide Johnson et al., 1996 from MERCK. The intracellular βIV5-3 peptide (CKLFIMN) and εV1-2 peptide (EAVSLKPT), both < 40 amino acids, were designed and tested by the Mochly-Rosen Lab (Johnson et al., 1996; Stebbins and Mochly-Rosen, 2001). DMSO was used as vehicle. Once inside nerve terminals—the only NMJ component that expresses PKCβI and PKCε- these peptides disrupt RACK-PKCβI or RACK-PKCε, respectively. Moreover, βIV5-3 peptide is connected via SS bond at the N terminus to the N terminal Cys of a deliverer peptide (CYGRKKRRQRRR, MW 2529), which enhances its cell-penetration. Working concentration was optimized to 100 μM for βIV5-3 peptide (Hurtado et al., 2017b) and 100 μM for εV1-2 peptide (Obis et al., 2015a).

### Presynaptic electrical stimulation of muscles

Diaphragm muscles were excised through the phrenic nerve, as it has been previously described (Besalduch et al., 2010; Obis et al., 2015a). From each animal, one hemidiaphragm underwent the experimental condition while the other was used as a control. Briefly, each hemidiaphragm was placed in oxygenated Ringer's solution (in mM: NaCl 137, KCl 5, CaCl_2_ 2, MgSO_4_ 1, NaH_2_PO_4_ 1, NaHCO_3_ 12, and glucose 12.1) and continuously bubbled with 95/5% of O_2_/CO_2_ at room temperature. Muscles were stimulated *ex vivo* through the phrenic nerve at 1 Hz by the A-M Systems 2100 isolated pulse generator (A-M System, Carlsborg, WA). The frequency of 1 Hz allows the maintenance of different tonic functions (e.g., PKC activation) without inducing synaptic plasticity. Visible contractions of the diaphragm muscle served to verify successful nerve stimulation resulting in contraction. Three main experiments were performed to distinguish the effects of synaptic activity from those of muscle activity (Table 1). In Experiment #1, to assess synaptic activity, we compared presynaptically stimulated muscles with contraction blocked by μ-CgTx-GIIIB with non-stimulated muscles also incubated with μ-CgTx-GIIIB to control for nonspecific effects of the blocker. In Experiment #2, to assess the effect of muscle contraction *per se*, we compared stimulated/contracting muscles with stimulated/non-contracting muscles. In Experiment #3, to assess the complete effect of synaptic activity with the resulting muscle contraction, we compared stimulated/contracting muscles with non-stimulated muscles, without exposure to μ-CgTx-GIIIB. Stimulation was performed during 30 min unless otherwise noted; e.g., sometimes it was stimulated for shorter times (10–30 s, 1–10 min).

**Table 1:**
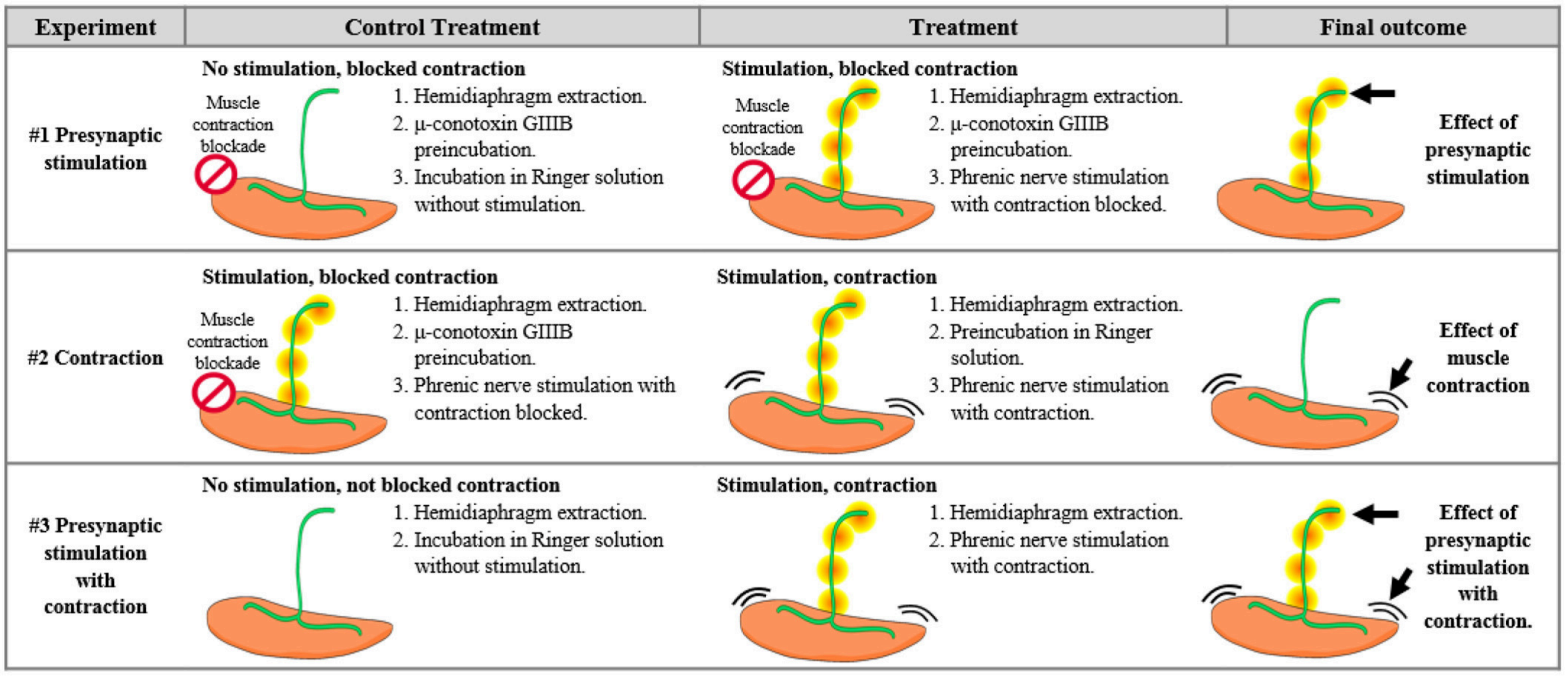
Differential effects of synaptic activity from those of muscle contraction.

*“Synaptic activity includes the presynaptic events related with nerve stimulation (1 Hz, 30 min), synaptic transmission and endplate potential generation due to ACh signaling (referred to as the Stimulation condition in the figures). Muscle contraction includes membrane depolarization of the muscle fiber involving voltage-dependent sodium channels and the resulting myofiber contraction (referred to as the Contraction condition in the figures). Finally, presynaptic Stimulation with Contraction treatment comprises the effects of synaptic activity and muscle contraction, showing complete neuromuscular activity*.”

*The Table has been adapted from Table 1 in the original article “[Muscle Contraction Regulates BDNF/TrkB Signaling to Modulate Synaptic Function through Presynaptic cPKCα and cPKCβI] by [Erica Hurtado, Víctor Cilleros, Laura Nadal, Anna Simó, Teresa Obis, Neus Garcia, Manel Santafé, Marta Tomàs, Katherine Halievski, Cynthia Jordan, Maria Angel Lanuza, Josep Tomàs]” The original article is an open access article distributed under the terms of the Creative Commons Attribution License (http://creativecommons.org/licenses/by/2.0), which permits unrestricted use, distribution, and reproduction in any medium, provided the original work is properly cited)*.

### Western blot

After dissection, muscles were immediately frozen in liquid nitrogen, and stored at −80°C. The muscles were homogenized using a high-speed homogenizer (overhead stirrer, VWR International, Clarksburg, MD) in lysis buffer [in mM:150 NaCl, 20 Tris-HCl (pH 7.4), 5 EDTA 1 PMSF, 50 NaF 1 OrtoVaNa, 1% Igepal CA-630, 1% Triton X-100, and protease inhibitor cocktail (1/100) (Sigma-Aldrich Corp., Saint Louis, MO, USA)] and protein lysates were collected. Protein concentrations were determined using the Bio-Rad DC protein assay (Bio-Rad, Hercules, CA).

Membrane/cytosol fractioning required muscle samples to be homogenized before freezing and in a detergent-free lysis buffer (in mM): 150 NaCl, 20 Tris-HCl (pH 7.4), 5 EDTA, 1 PMSF, 50 NaF, 1 OrtoVaNa, and protease inhibitor cocktail (1/100). Homogenized samples were centrifugued at 1,000 g for 15 min and the resulting supernatant was further centrifuged at 130,000 g for 1 h. Afterwards, the supernatant corresponded to the cytosolic fraction and the pellet, to the membrane fraction. The membrane was resuspended in lysis buffer containing (in mM) 150 NaCl, 20 Tris-HCl (pH 7.4), 5 EDTA, 1 PMSF, 50 NaF, 1 OrtoVaNa, and protease inhibitor cocktail (1/100). Glyceraldehyde-3-phosphate dehydrogenase (GAPDH) and Na^+^/K^+^-ATPase immunoreactivity were used to determine the purity of the fractionation. Protein concentration was determined using the Bio-Rad DC protein assay (Bio-Rad, Hercules, CA).

Protein samples of 30 μg were separated by 8 or 15% SDS-polyacrylamide electrophoresis and electrotransferred to PVDF membranes (Hybond™-P; Amersham, GE Healthcare). Membranes were blocked in tris-buffered saline with Tween 20 containing 5% (W/V) nonfat dry milk or 5% (W/V) bovine serum albumin and incubated with a primary antibody overnight at 4°C and the corresponding secondary antibody for 1 h.

Blots were visualized with the ChemiDoc XRS+ Imaging System (Bio-Rad, Hercules, CA) and the enhanced chemiluminescence (ECL) kit from Amersham Life Science, Arlington Heights, IL. Sample loading and antibodies were optimized to guarantee the linear range during the first minute of exposure. The densitometry of the bands was obtained with the MetaMorph Microscopy Automation and Image Analysis Software (RRID: SCR_002368). The integrated optical density of the bands was normalized in relation to (1) the background values and (2) the total protein transferred on PVDF membranes, measured by total protein blot staining (Sypro Ruby, Bio-Rad). The relative variations between the experimental samples and the control samples were calculated from the same membrane image. Data are mean values ± SEM. Statistical significance of the differences between groups was evaluated under the Wilcoxon test or the Student's *t*-test and the normality of the distributions was tested with the Kolmogorov–Smirnov test. The criterion for statistical significance was *p* < 0.05 vs. the control.

### Immunohistochemistry and confocal microscopy

Whole muscles were processed by immunohistochemistry to detect and localize Munc18-1 at the NMJ. Diaphragm and LAL muscles from young adult rats were fixed with 4% paraformaldehyde for 30 min. After fixation, the muscles were rinsed with phosphate buffer saline (PBS) and incubated in 0.1 M glycine in PBS. The muscles were permeabilized with 0.5% Triton X-100 in PBS, and nonspecific binding was blocked with 4% BSA. Then, they were incubated overnight at 4°C in mixtures of three primary antibodies raised in different species (anti-Munc18-1; anti-syntaxin; anti-neurofilament-200 to label the axon terminal and anti-S-100 to label Schwann cells) and then rinsed. The muscles were then incubated for 4 h at room temperature in a mixture of appropriate secondary antibodies. AChRs were detected with α- Bungarotoxin conjugated to tetramethylrhodamine (TRITC). As a control, primary antibodies were omitted from some muscles during the immunohistochemical procedures. These control muscles never exhibited positive staining. In double-staining protocols, omitting either one of the two primary antibodies completely abolished the corresponding staining and there was no cross-reaction with the other primary antibody. At least three muscles were used as negative controls. Localization of the Munc18-1 at the NMJ was observed with a laser-scanning confocal microscope (Nikon TE2000-E). Special consideration was given to avoid contamination between channels. In experiments involving negative controls, the photomultiplier tube gains and black levels were identical to those used for a labeled preparation made in parallel with the control preparations. At least 6 muscles and 25 endplates per muscle were studied. Zeiss LSM880 AiryScan Confocal microscope, with higher resolution, was used to image three muscles treated with PMA or εV1-2 or PBS and doubly immunolabeled to detect Munc18-1 and AChR. Images were taken with a Zeiss PlanApo × 63 1.42 NA oil objective. The fluorescence intensity of each NMJ, normalized to background, was analyzed with ImageJ (ImageJ, RRID:SCR_003070) under identical conditions of light intensity and camera gain settings. Images were assembled using Adobe Photoshop software (Adobe Systems, San Jose, CA; RRID:SCR_014199) and neither the contrast nor brightness were modified.

## Results

### Munc18-1 and pMunc18-1 in adult skeletal muscle

**Figure 1:**
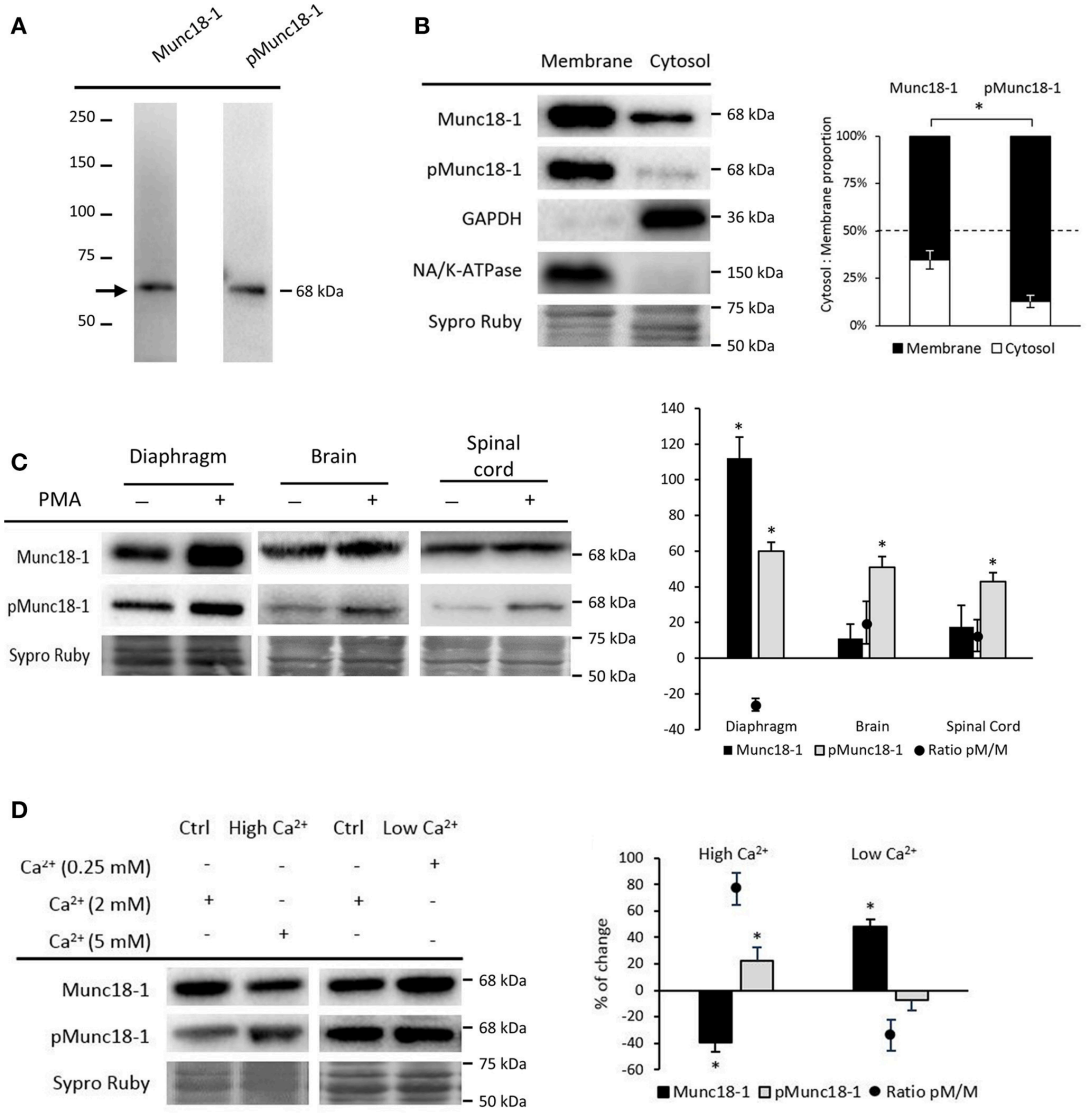
Munc18-1 and pMunc18-1 in adult skeletal muscle under basal conditions. **(A)** Representative Western blot bands from diaphragm showing specificity for the antibodies Munc18-1 and pMunc18-1 (Ser-313). **(B)** Representative Western blot bands and quantification of membrane and cytosol distribution of Munc18-1 and pMunc18-1. Both are mainly located in the membrane but pMunc18-1 is barely detectable in the cytosolic fraction. GAPDH is exclusively in the cytosol fraction and the Na^+^/K^+^-ATPase is in the membrane. **(C)** Representative Western blot bands and quantification of Munc18-1 and pMunc18-1 after PMA treatment in diaphragm, brain and spinal cord. **(D)** Representative Western blot bands and quantification of Munc18-1 and pMunc18-1 after different calcium concentrations. High Ca^2+^ concentration increases pMunc18-1 levels while decreases Munc18-1. Data are mean percentage ± SEM, ^*^p < 0.05 (*n* = 5).

The presence of Munc18-1 and pMunc18-1 (Ser-313) was determined in diaphragm, brain and spinal cord by immunobloting. In all tissues, the used antibodies only recognized the corresponding protein, reacting with a 70 kDa band for Munc18-1 and a 68 kDa band for pMunc18-1 as predicted by the manufacturers (Figure 1A).

Munc18-1 phosphorylation regulates its interaction and dissociation with syntaxin-1 and other binding partners in the membrane (Cijsouw et al., 2014). Therefore, we analyzed Munc18-1 and pMunc18-1 in cytosol and membrane fractions under basal conditions (Figure 1B). Results showed that Munc18-1 is distributed in both cytosol and membrane fractions, with higher presence in the latter. On the other hand, pMunc18-1 is strongly linked to the membrane and less detectable in the cytosol. We validated cytosol and membrane fractioning with GAPDH and Na^+^/K^+^-ATPase immunoblotting. GAPDH was detected in the cytosol fraction and essentially undetectable in the membrane fraction. In concordance, the transmembrane protein Na^+^/K^+^-ATPase was highly enriched at the membrane and undetectable in the cytosol.

Diaphragm muscles in basal conditions (without nerve stimulation) showed a remarkable amount of Munc18-1 and a particulary high amount of its phosphorylated form on Ser-313 (Figure 1C). The spinal cord and brain showed quite high amounts of Munc18-1 but pMunc18-1 was barely detectable as previously published in resting neurons and cromaffin cells (de Vries et al., 2000; Barclay et al., 2003; Craig et al., 2003). Ser-313 phosphorylation of Munc18-1 occurs in response to PKC stimulus *in vitro* (Fujita et al., 1996; de Vries et al., 2000; Barclay et al., 2003). Therefore, we investigated whether activation of PKC alters pMunc18-1 levels. Figure 1C shows that both Munc18-1 and its phosphorylation are strongly increased in diaphragm by the pan-PKC activator PMA treatment (10 nM, 30 min), although the phosphorylation ratio pMunc18-1/Munc18-1 did not change. This was unexpected as PMA strongly increases Munc18-1 phosphorylation in neurons and chromaffin cells. Accordingly, we found that PMA increased pMunc18-1 levels without modifying total Munc18-1 in all central nervous system (CNS) tissues analyzed, being their phosphorylation ratios increased.

It has been shown that Ca^2+^, the primary intracellular trigger for exocytosis, induces Munc18-1 phosphorylation on Ser-313 in chromaffin cells (Craig et al., 2003). Here, we determined whether Ca^2+^ also modulates the phosphorylation at the NMJ. Figure 1D shows that high Ca^2+^ (5 mM, 30 min) increases pMunc18-1 and decreases total Munc18-1. Accordingly, the ratio pMunc18-1/Munc18-1 increases (76.68% ± 12.04, *p* < 0.05). On the contrary, low Ca^2+^ (0.25 mM, 30 min) only increases total Munc18-1 (47.89% ± 5.81; *p* < 0.05), decreasing the ratio pMunc18-1/Munc18-1 (36.28% ± 10.85, *p* < 0.05). Therefore, the data show that expression and phosphorylation of Munc18-1 on Ser-313 depend on Ca^2+^ at the NMJ in a similar way than in synaptosomes, chromaffin cells and neuron cultures (Craig et al., 2003; Wierda et al., 2007).

### Munc18-1 is located in the nerve terminal at the neuromuscular junction

**Figure 2:**
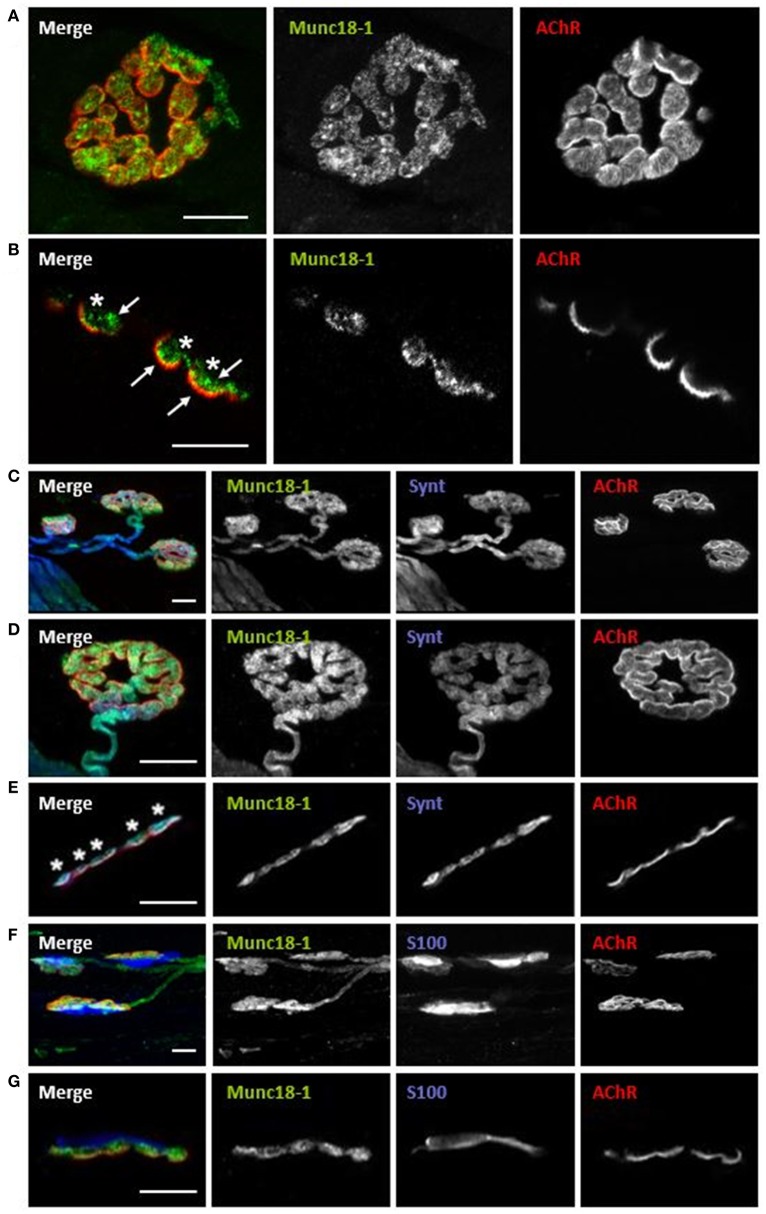
Munc18-1 is exclusively located in the presynaptic component of the NMJ. Multiple-immunofluorescence-stained muscles visualized at the confocal microscope. **(A)** Munc18-1 colocalizes with AChR from a NMJ en face view. **(B)** Munc18-1 colocalizes with AChR from a NMJ en side view. **(C,D)** Munc18-1 colocalizes with syntaxin and AChR from en face view. **(E)** Munc18-1 colocalizes with syntaxin and AChR from en side view. **(F)** Munc18-1 colocalizes with S-100 and AChR from en face view. **(G)** Munc18-1 colocalizes with S-100 and AChR from a NMJ en side view. Scale bars = 10 μm.

Immunofluorescence staining coupled with confocal microscopy analysis was performed to determine the location of Munc18-1 at the adult NMJ. Experiments were performed in the diaphragm and LAL muscles and immunoreactivity for Munc18-1 was identical in both muscles. We colocalize Munc18-1 with NMJ markers (syntaxin for the axon and presynaptic terminal, S-100 for the Schwann cell and AChR for the postsynaptic membrane in the myocyte). All pictures in Figure 2 show intense immunoreactivity for Munc18-1 in the synaptic area, identified with AChR labeling. Figures 2A,B show two NMJs with double labeling: AChRs in red and Munc18-1 in green. The *en face* (Figure 2A) and the *en side* (Figure 2B) images show Munc18-1-positive green immunolabeling concentrated at the presynaptic position, over the red postsynaptic gutters (asterisks in Figure 2B). Munc18-1 labeling has stronger concentration in some areas (arrows). Figures 2C–G show NMJs with triple labeling: AChRs in red, nerve terminals (syntaxin; Figures 2C–E) or Schwann cells (S-100; Figures 2F,G) in blue and Munc18-1 in green. Figures 2C,F show low magnification images reflecting an abundant Munc18-1-positive green immunolabeling concentrated at the synapses. Figures 2D,E show detailed Munc18-1-positive green immunolabeling concentrated at the presynaptic position over the red postsynaptic gutters and colocalized with syntaxin (Figure 2E, *en side* NMJ, asterisks). Figures 2F,G show that there is not colocalization between Munc18-1 and the Schwann cell. Finally, in Figures 2C,D,F it appears that pre-terminal axon is also Munc18-1-positive. Thus, Munc18-1 is located in the nerve terminal at the neuromuscular junction.

### Synaptic activity increases Munc18-1 phosphorylation in the NMJ

**Figure 3:**
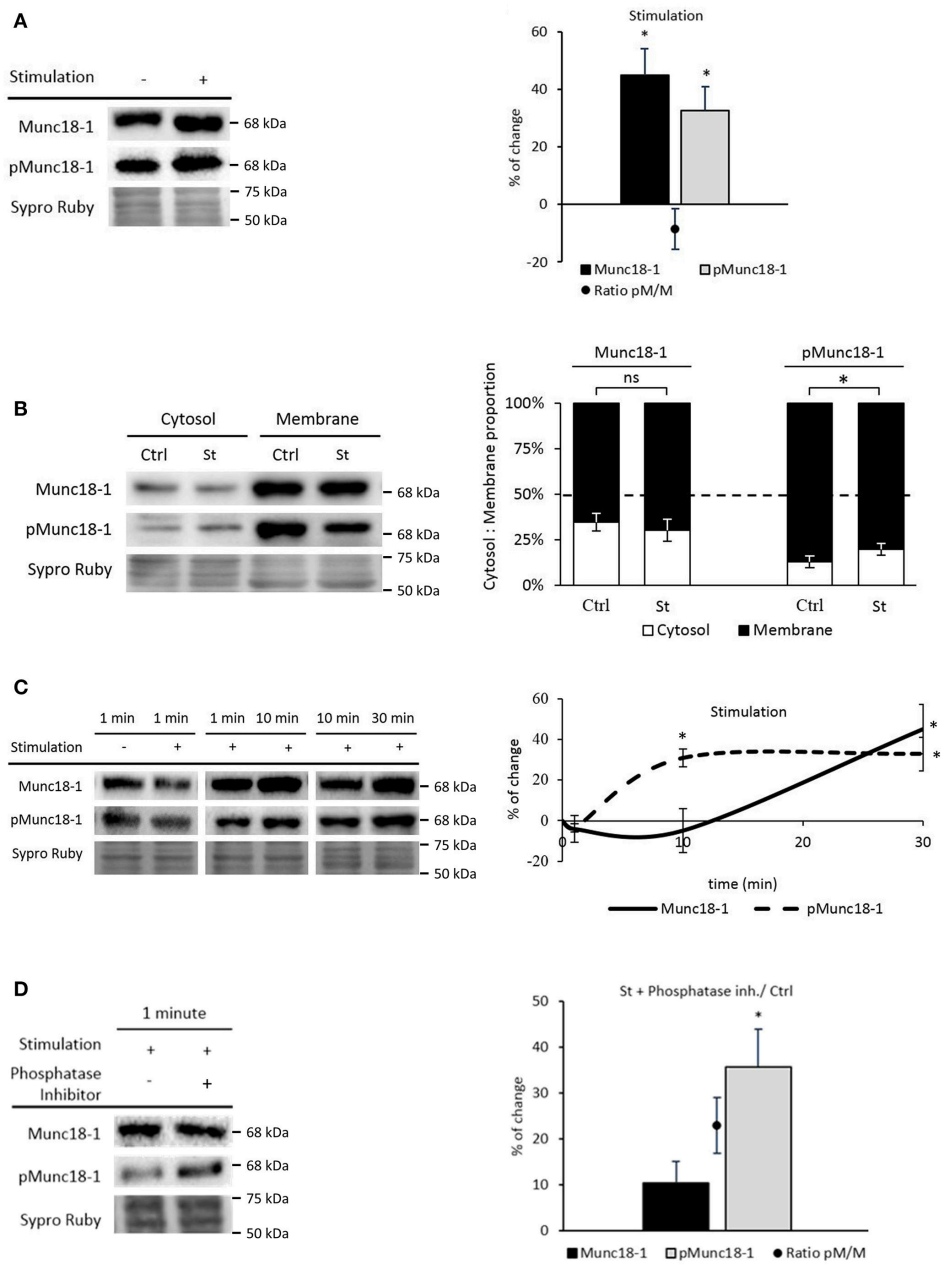
Synaptic activity increases Munc18-1 phosphorylation in the NMJ. **(A)** Representative Western blot bands and quantification show that both Munc18-1 and pMunc18-1 increase after stimulation without contraction regarding to basal conditions. **(B)** Representative Western blot bands and quantification show that pMunc18-1 increases in the cytosol fraction while Munc18-1 does not change after stimulation without contraction regarding to basal conditions. **(C)** Representative Western blot bands and their representation show Munc18-1 and pMunc18-1 modulation through different stimulation times without contraction. While Munc18-1 progressively increases from 10 to 30 min, pMunc18-1 reaches its maximum at 10 min, which is sustained until 30 min. **(D)** Representative Western blot bands and quantification show Munc18-1 and pMunc18-1 after 1 min of presynaptic stimulation treatment preincubated with phosphatase inhibitors. The pMunc18-1 levels and pMunc18-1/Munc18-1 ratio significantly increase. Data are mean percentage ± SEM, ^*^p < 0.05 (*n* = 5).

Nerve activity and the resulting muscle contraction regulate presynaptic PKC, whose activity may be related with Munc18-1 phosphorylation at the NMJ (Besalduch et al., 2010; Obis et al., 2015a; Hurtado et al., 2017b). Therefore, we first isolated the effect of the presynaptic stimulation (and synaptic transmission) from the effect of the muscle cell contraction, by performing experiments in which contraction was inhibited (Table 1). Synaptic activity includes the presynaptic events related with nerve stimulation (1 Hz, 30 min), synaptic transmission and endplate potential generation due to ACh signaling [termed *Stimulation (St)* in the figures]. Muscle contraction includes membrane depolarization of the muscle fiber involving voltage-gated sodium channels and myofiber contraction (termed *Contraction* in the figures). In particular, muscle contraction was inhibited using μ-CgTx-GIIIB (Obis et al., 2015a,b) that preserves neurotransmission. The results show that nerve stimulation significantly increased Munc18-1 (45.06% ± 9.26, *p* < 0.05) and pMunc18-1 (32.89% ± 8.30, *p* < 0.05) levels (Figure 3A). In concordance, the ratio pMunc18-1/Munc18-1 remained unchanged (8.52% ± 7.02, *p* > 0.05). Because the analysis of protein translocation is important to understand their functionality, we proceeded to evaluate whether the stimuli can move Munc18-1 between membrane and cytosol. Nerve stimulation significantly increased pMunc18-1 in the cytosol fraction (53.91% ± 3.07, *p* < 0.05; Figure 3B), indicating that stimulation-induced phosphorylation results in a moderate disconnection of the molecule from the membrane.

We analyzed Munc18-1 and pMunc18-1 at shorter times of stimulation (1 and 10 min, 1 Hz) because phosphorylation is a rapid process. Surprisingly, 1 min of presynaptic stimulation did not modify pMunc18-1 levels while 10 min significantly increased them (30.81% ± 4.41, *p* < 0.05) to match the effect of 30 min of stimulation. On the other hand, Munc18-1 gradually increased from 10 to 30 min (Figure 3C).

To understand why pMunc18-1 did not increase after 1 min stimulation, we analyzed pMunc18-1 levels after phosphatase activity inhibition (since several phosphatases regulate synaptic activity at the NMJ, see Hurtado et al., 2017b). Without phosphatases, 1 min stimulation significantly increased pMunc18-1 levels (35.68% ± 8.13, *p* < 0.05; Figure 3D) indicating that phosphatase activity regulates Munc18-1 phosphorylation-dephosphorylation dynamics at short stimulation times.

### nPKCε and cPKCβI activities regulate Munc18-1 phosphorylation in the NMJ

**Figure 4:**
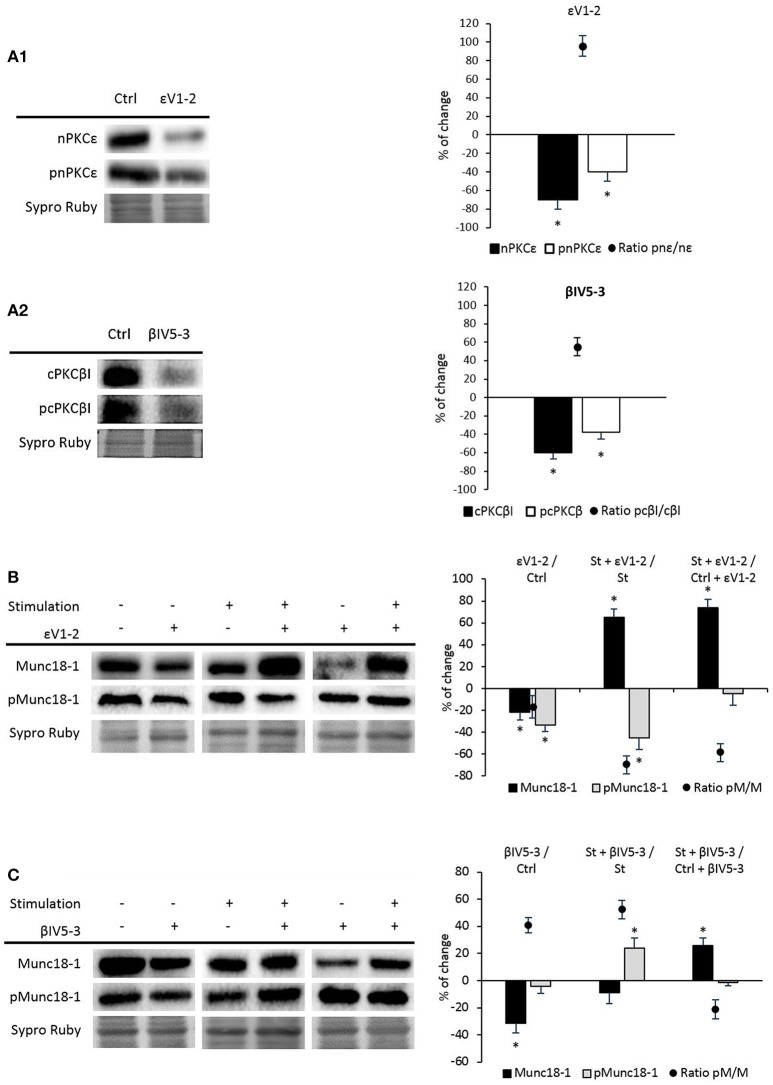
nPKCε and cPKCβI regulate synaptic activity-induced Munc18-1 phosphorylation. **(A1,A2)** Representative Western blot bands and quantification show that εV1-2 and βIV5-3 peptides inhibit the presence of nPKCε and cPKCβI and their phosphorylation levels under basal conditions. **(B)** Representative Western blot bands and quantification show that under basal conditions both Munc18-1 and pMunc18-1 significantly decrease (εV1-2/Ctrl: muscles in basal conditions vs. basal conditions preincubated with εV1-2 peptide). Moreover, in both (St + εV1-2/ St: synaptic activity compared with the εV1-2 peptide) and (St + εV1-2/ Ctrl + εV1-2: basal conditions compared with stimulated samples; both treated with εV1-2 peptide) pMunc18-1/Munc18-1 ratios significantly decrease. **(C)** Representative Western blot bands and quantification show that in both (βIV5-3/Ctrl: muscles in basal conditions vs. preincubated with βIV5-3 peptide) and (St+βIV5-3/St: synaptic activity compared with the βIV5-3 peptide) conditions, the ratio of pMunc18-1/Munc18-1 significantly increases. Moreover, in (St+βIV5-3/Ctrl+βIV5-3: basal conditions compared with stimulated samples; both treated with βIV5-3 peptide) pMunc18-1/Munc18 ratio is significantly decreased. Data are mean percentage ± SEM, ^*^p < 0.05 (*n* = 5).

nPKCε and cPKCβI isoforms are good candidates to regulate the phosphorylation of Munc18-1 as they are exclusively located in the nerve terminal of the NMJ, regulated by synaptic activity and involved in neurotransmitter release (Besalduch et al., 2010; Obis et al., 2015a,b; Hurtado et al., 2017b). In order to test whether these two isoforms are involved in the phosphorylation of Munc18-1, muscles were incubated with the nPKCε-specific translocation inhibitor peptide, epsilon V1-2 (εV_1−2_; Johnson et al., 1996), and with the cPKCβI-specific translocation inhibitor peptide, betaI V5-3 (βIV_5−3_; Liu et al., 1999; Zhang et al., 2015). βIV_5−3_ peptide is derived from the V5 domain of cPKCβI and binds to the anchoring protein βI-RACK, disrupting the interaction between both proteins, impairing cPKCβI translocation to the membrane and its activation. Similarly, the εV_1−2_ peptide derives from the V1 domain of nPKCε and also binds to its RACK receptor. We previously demonstrated that εV_1−2_ and βIV_5−3_ peptides inhibit the presence of pnPKCε and pcPKCβI in the membrane fraction of the diaphragm, respectively (Obis et al., 2015a,b; Hurtado et al., 2017b). Accordingly, Figure 4A1 shows that the peptide εV_1−2_ decreases nPKCε and pnPKCε protein levels and Figure 4A2 shows that the peptide βIV_5−3_ decreases cPKCβI and pcPKCβI levels. Next, we determined how nPKCε and cPKCβI activities affect Munc18-1 in basal conditions and after presynaptic stimulation.

### nPKCε

**Figure 5:**
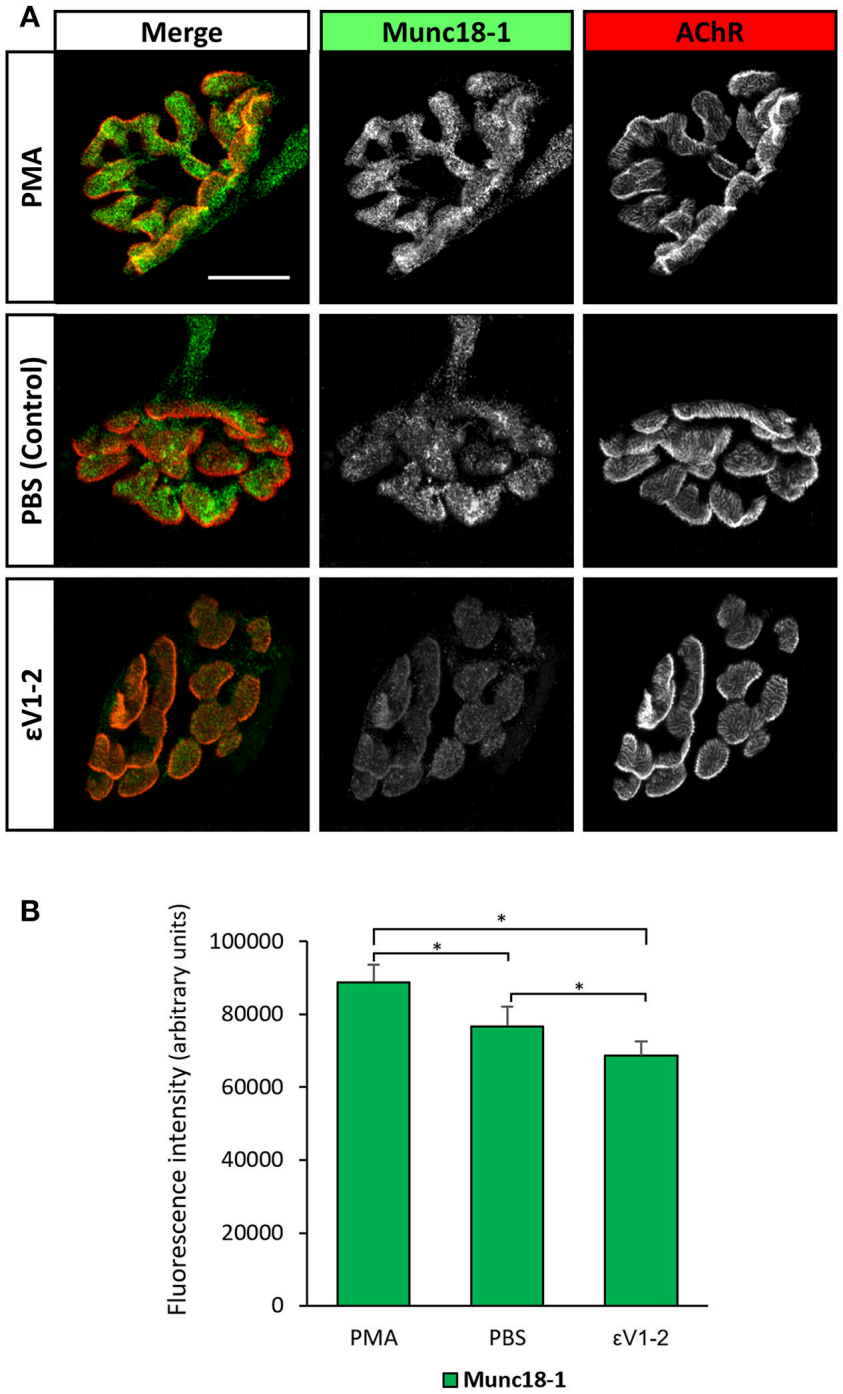
Munc18-1 expression at the nerve terminal is regulated by PKC activity. **(A)** Representative confocal doubly-immunofluorescence-stained NMJs images (Munc18-1 and AChR) after a treatment with PBS as a control, PMA, and εV1-2 peptide. PMA increases Munc18-1 fluorescence intensity while εV1-2 peptide decreases it. Scale bar = 10 μm. **(B)** Fluorescence intensity quantification of Munc18-1. Data are mean value ± SEM, ^*^p < 0.05.

Figure 4B shows that the εV_1−2_ peptide significantly decreases Munc18-1 and pMunc18-1. This indicates that, in basal conditions at the skeletal muscle, nPKCε regulates Munc18-1 levels and, maybe consequently, its phosphorylation, suggesting that nPKCε could have a positive tonic effect regulating pMunc18-1. The ratio pMunc18-1/Munc18-1 in basal conditions was not significantly decreased (−16.71% ± 9.82, *p* > 0.05). Higher resolution NMJ images after a treatment with PBS as a control, PMA and εV_1−2_ peptide indicate that when all the PKC isoforms are activated by PMA, Munc18-1 levels are upregulated in the nerve terminal (see Figure 5), as previously stated. Furthermore, εV_1−2_ peptide downregulates Munc18-1 levels in the nerve terminal and confirms Western blot results. PBS did not reveal differences with non-treated animals.

To study the effects of synaptic activity on nPKCε activity, we stimulated (1 Hz, 30 min) phrenic nerves of muscles previously incubated with the blocking peptide (100 μM, 30 min). We found a significant decrease in pMunc18-1 level (−44.96% ± 10.80; *p* < 0.05) and a significant increase in total Munc18-1 level (65.32% ± 7.63; *p* < 0.05). In concordance, the ratio pMunc18-1/Munc18-1 was significantly decreased (−69.82% ± 8.03; *p* < 0.05) indicating that nPKCε specifically enhances phosphorylation of Munc18-1 when synaptic activity increases (Figure 4B).

Furthermore, to know whether nPKCε was responsible of the increase of pMunc18-1 after nerve stimulation, we compared control and stimulated samples in which nPKCε was blocked (*St* + ε*V*_1−2_ vs*. Ctrl* + ε*V*_1−2_). We found a significant increase of Munc18-1 (73.63 ± 7.90; *p* < 0.05) without changes on pMunc18-1 level (−4.49 ± 10.74; *p* > 0.05; Figure 4B). These results show that nPKCε induces Munc18-1 phosphorylation during synaptic activity and reinforce the idea that nPKCε downregulates Munc18-1 levels, induced by the nerve stimulation.

### cPKCβI

We also analyzed the effect of blocking the membrane translocation of cPKCβI with the βIV_5−3_ peptide. Figure 4C shows that the βIV_5−3_ peptide decreased total Munc18-1 levels but did not change pMunc18-1 in basal conditions (ratio pMunc18-1/Munc18-1: 40.92 ± 5.68; *p* < 0.05). However, βIV_5−3_ during synaptic activity caused a significant increase in pMunc18-1 with no change in total Munc18-1. In concordance, the ratio pMunc18-1/Munc18-1 was significantly increased (52.35 ± 7.21; *p* < 0.05), indicating that the role of the cPKCβI during synaptic activity reduces Munc18-1 phosphorylation. As with nPKCε above, we compared control and stimulated samples in which cPKCβI had been blocked (Figure 4C). We found a significant increase of Munc18-1 (26.01 ± 5.41; *p* < 0.05) with no changes in pMunc18-1 (−1.20 ± 3.35; *p* > 0.05) being the ratio pMunc18-1/Munc18-1 (−21.18 ± 6.74; *p* < 0.05). These results indicate that cPKCβI is necessary for the increase of Munc18-1 phosphorylation caused by nerve stimulation but not for the increase of Munc18-1 levels (Figure 4C, last column).

**Figure 6:**
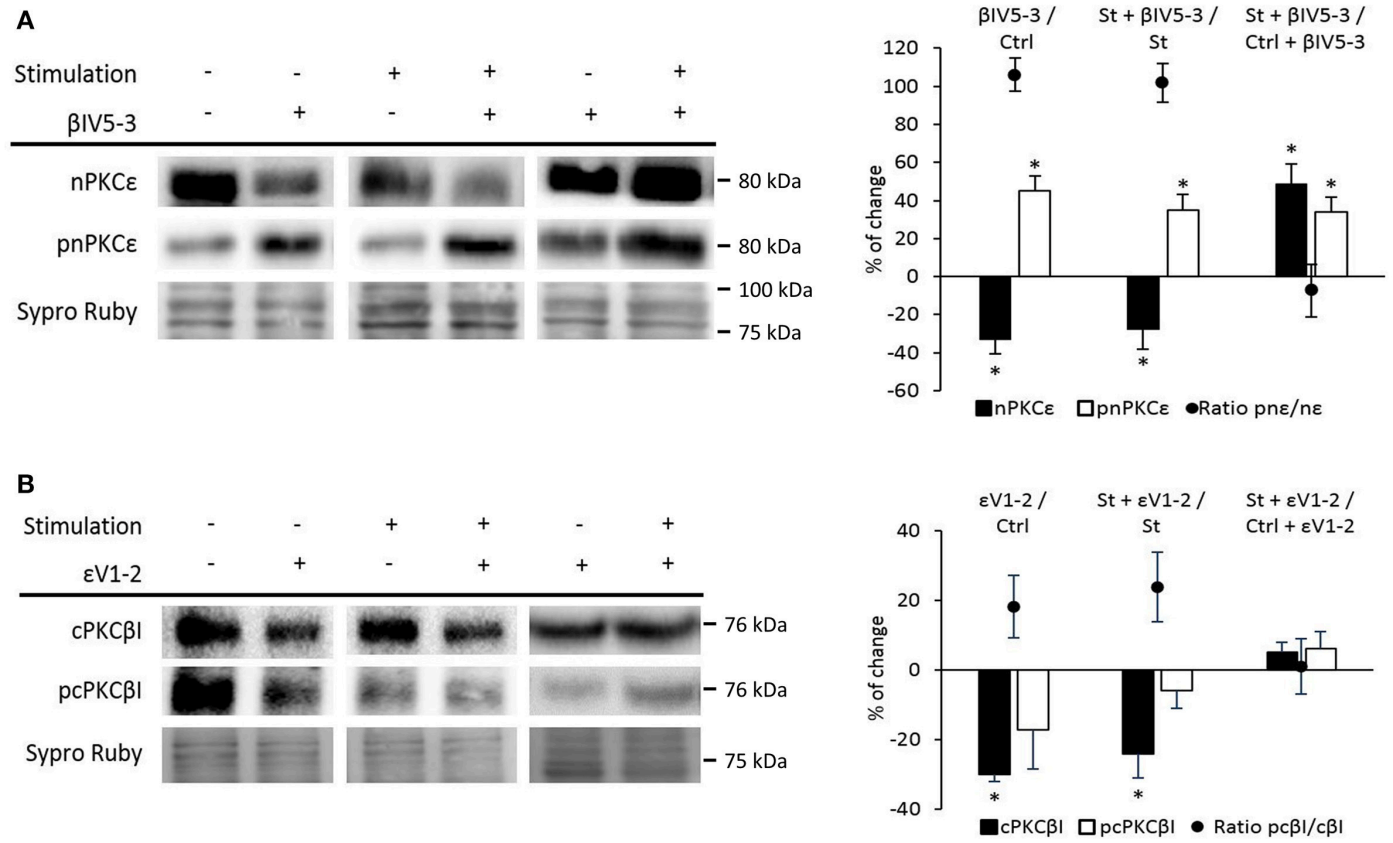
Relation between nPKCε and cPKCβI. **(A)** Representative Western blot bands and quantification show that both (βIV5-3/Ctrl: muscles in basal conditions vs. preincubated with βIV5-3 peptide) and (*St*+β*IV5-3/St*: synaptic activity compared with the βIV5-3 peptide), the ratio pnPKCε/nPKCε significantly increases. Moreover, in (*St*+β*IV5-3/Ctrl*+β*IV5-3***:** basal conditions compared with stimulated samples; both treated with βIV5-3 peptide) both nPKCε and pnPKCε significantly increase. **(B)** Representative Western blot bands and quantification show that in both (ε*VI-2/Ctrl*: muscles in basal conditions vs. preincubated with εVI-2 peptide) and (*St*+ε*VI-2/St*: synaptic activity compared with the εVI-2 peptide) the cPKCβI total levels significantly decreased, being the ratio pcPKCβI/cPKCβI in this last condition significantly increased. Data are mean percentage ± SEM, ^*^p < 0.05 (*n* = 5).

Having established that nPKCε and cPKCβI oppositely regulate Munc18-1 phosphorylation, we next investigated whether cPKCβI and nPKCε regulate each other. Figure 6A shows that βIV_5−3_ was able to significantly increase pnPKCε protein levels (with a significant decrease in nPKCε) in both basal and synaptic activity conditions. The pnPKCε/nPKCε ratio significantly increased (basal conditions: 106.06 ± 8.69; *p* < 0.05; synaptic activity: 101.84 ± 10.45; *p* < 0.05). Moreover, in St + βIV_5−3_ vs. Ctrl + βIV_5−3_ both nPKCε and pnPKCε significantly increased. These results indicate that cPKCβI isoform downregulates nPKCε phosphorylation and maybe its activity. Together, these data indicate that synaptic activity activates pnPKCε to phosphorylate Munc18-1 while cPKCβI decreases it, maybe inhibiting pnPKCε.

On the contrary, Figure 6B shows that εV_1−2_ significantly decrease cPKCβI without changing pcPKCβI levels in both basal and synaptic activity conditions. These results indicate that pnPKCε isoform enhances cPKCβI levels but does not affect its activity. Together, these results demonstrate a mutual regulatory influence between nPKCε and cPKCβI.

### BDNF/TrkB pathway regulates pMunc18-1 in the NMJ

**Figure 7:**
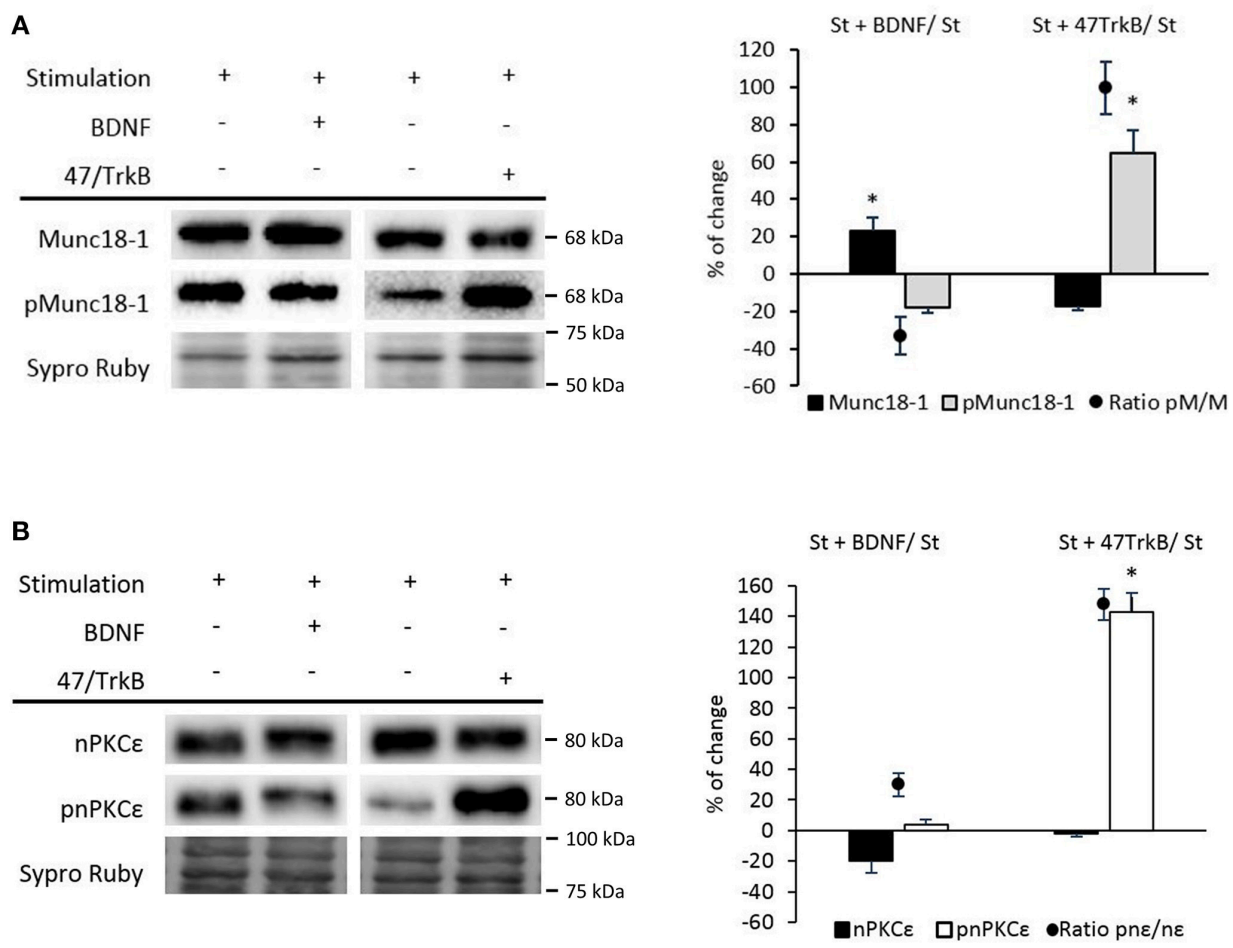
BDNF/TrkB signaling modulates Munc18-1, pMunc18-1, nPKCε, and pnPKCε levels under synaptic activity conditions. **(A)** Representative Western blot bands and quantification show that after the treatment with BDNF, the ratio of pMunc18-1/Munc18-1 significantly decreases while after the 47/TrkB treatment, it significantly increases. **(B)** Representative Western blot bands and quantification show that after the 47/TrkB treatment, pnPKCε significantly increases without affecting nPKCε levels. In accordance, the ratio is significantly increased. Data are mean percentage ± SEM, ^*^p < 0.05 (*n* = 5).

We recently described that synaptic activity enhances BDNF/TrkB/cPKCβI activity (Hurtado et al., 2017b). To demonstrate whether TrkB affects pMunc18-1, we suppressed TrkB activity in nerve-stimulated diaphragm using the anti-TrkB antibody 47/TrkB which is a selective TrkB inhibitor (Balkowiec and Katz, 2000). In stimulated muscles, TrkB blockade significantly increased pMunc18-1 without affecting Munc18-1 levels (Figure 7A). In concordance, the ratio pMunc18-1/Munc18-1 significantly increased (99.69 ± 14.04; *p* < 0.05). This result indicates that endogenous BDNF-TrkB pathway inhibits Munc18-1 phosphorylation. However, exogenous BDNF (10 nM, 30 min) in nerve-stimulated muscles (*St*) did not modify the pMunc18-1 levels during stimulation (Figure 7A). These results show that, endogenous -but not exogenous- BDNF acts through TrkB to inhibit Munc18-1 phosphorylation. On the contrary, exogenous BDNF slightly increased Munc18-1 protein levels, perhaps inducing its synthesis or decreasing its degradation. BDNF concentration was optimized in a previous dose—response and time-course study in the same muscle model to determine its effect on the size of the evoked end-plate potential (EPPs) (Garcia et al., 2010). Synaptic activity-induced TrkB effects are similar to those of cPKCβI suggesting that cPKCβI and TrkB work coordinately to regulate the phosphorylation of Munc18-1 in this condition. It is unknown whether BDNF/TrkB signaling regulates synaptic activity-induced nPKCε activity. Therefore, we next tested how 47/TrkB affects nPKCε and pnPKCε during synaptic activity. Figure 7B shows that 47/TrkB significantly increased pnPKCε levels without affecting nPKCε, indicating that TrkB activity decreases the phosphorylation of nPKCε (ratio pnPKCε/nPKCε: 147.88 ± 10.12; *p* < 0.05) and probably its activity. Because TrkB decreases pMunc18-1 levels (Figure 7A), the decrease in pnPKCε may be related with a decrease in its activity. Together, these results are good evidence that endogenous BDNF -but not exogenous- inhibits, through TrkB, pnPKCε activity which is positively related to pMunc18-1 during presynaptic stimulation.

### Muscle contraction downregulates pMunc18-1 against the activity of nPKCε, cPKCβI, and BDNF/TrkB

It has been previously established that the presynaptic PKC isoforms ε and βI are differently regulated by nerve activity and the resulting muscle activity in the NMJ. Specifically, muscle activity *per se* enhances presynaptic nPKCε and cPKCβI protein levels suggesting a retrograde factor regulation from the muscle (Besalduch et al., 2010; Obis et al., 2015a,b). Therefore, we investigated the role of muscle contraction over Munc18-1 and pMunc18-1 protein levels and the relationship with these PKC isoforms.

**Figure 8:**
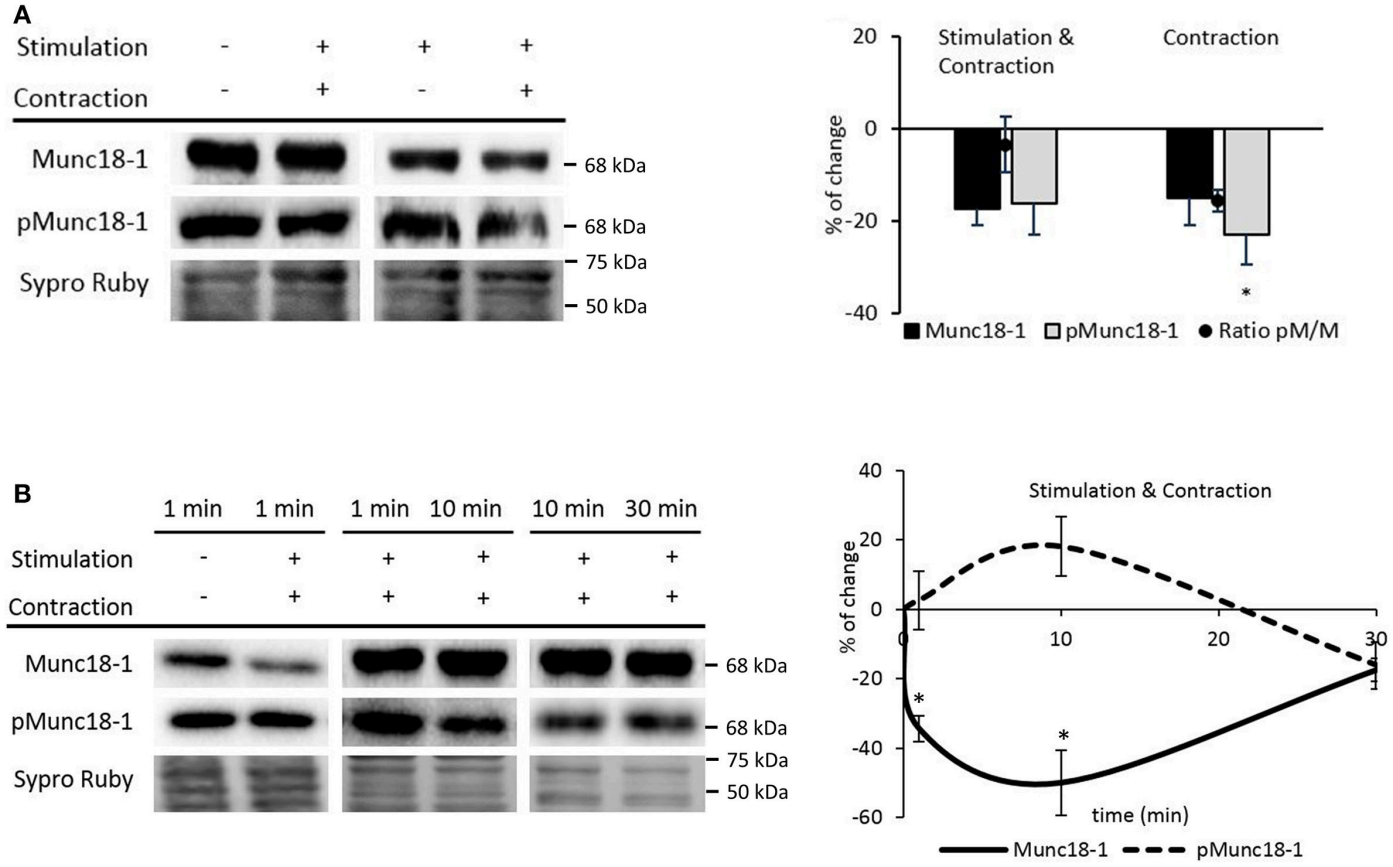
Muscle contraction modulates pMunc18-1 levels in the skeletal muscle. **(A)** Representative Western blot bands and quantification show a significant pMunc18-1 decrease after electrical stimulation at 1 Hz for 30 min resulting in contraction **(B)** Representative Western blot bands and the time course representation show that muscle contraction significantly increases pMunc18-1 at 10 min and significantly decreases Munc18-1 at 1 min and is maintained until 30 min. Data are mean percentage ± SEM, ^*^p < 0.05 (*n* = 5).

Figure 8A shows that electrical stimulation and contraction did not change Munc18-1 and pMunc18-1, although a non-significant decrease was detected. Interestingly, when the effect of muscle contraction was itself analyzed by comparing stimulated muscles with unaltered contraction (*Stimulation with Contraction*) with stimulated muscles that were preincubated with μ-CgTx-GIIIB (*Stimulation*), we found that pMunc18-1 levels decreased (−22.85 ± 6.50; *p* < 0.05) and the ratio of pMunc18-1/Munc18-1 was maintained (−15.48 ± 2.40; *p* > 0.05). These results indicate that muscle contraction *per se* prevents (or reverts to control values) the nerve activity-induced Munc18-1 and pMun18-1 increase (showed in Figure 3A).

We next tested whether muscle contraction prevents the nerve-induced phosphorylation of Munc18-1 even at short times of stimulation. Therefore, we performed experiments at 1, 10, and 30 min with nerve-electrical stimulation resulting in muscle contraction. Ten minutes of muscle contraction, slightly increased Munc18-1 phosphorylation (18.06% ± 8.52; *p* > 0.05) and declined under baseline at 30 min of stimulation-induced contraction (Figure 8B). On the contrary, 1 min of muscle contraction significantly decreased Munc18-1 levels (−34.41% ± 3.74; *p* < 0.05; Figure 8B), which further decreased at 10 min (−49.19 ± 7.02; *p* < 0.05) and raised back to the baseline after 30 min (−17.43% ± 3.35; *p* > 0.05; Figure 8B). When these results are compared with those in Figure 3C, the time course of pMunc18-1 between 1 and 10 min is similar with and without contraction, although lower with contraction. However, the effect of longer muscle contraction times (10–30 min) is to decrease pMunc18-1 protein levels. Together, these results indicate that muscle contraction only prevents pMunc18-1 after continuous stimulation.

**Figure 9:**
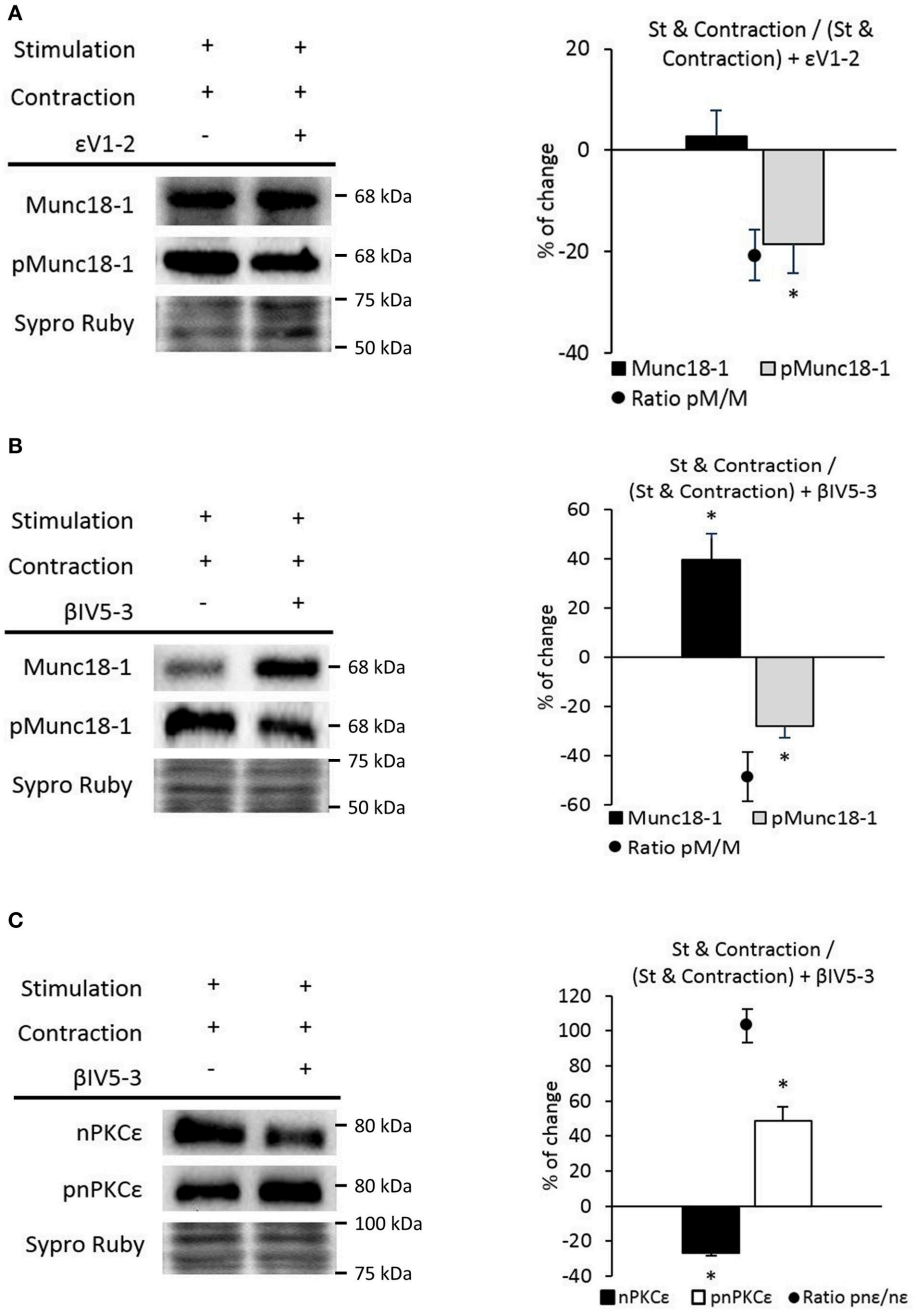
nPKCε and cPKCβI modulate Munc18-1 phosphorylation under muscle contraction conditions. **(A)** Representative Western blot bands and quantification show that Munc18-1 phosphorylation and the pMunc18-1/Munc18-1 ratio are significantly decrease after stimulation resulting in contraction in nPKCε inhibitor peptide εV1-2 preincubated muscles. **(B)** Representative Western blot bands and quantification show that the pMunc18-1/Munc18-1 ratio are significantly decreases after stimulation resulting in contraction in cPKCβI inhibitor peptide βIV5-3 preincubated muscles. **(C)** Representative Western blot bands and quantification show that the phosphorylation of nPKCε and the pnPKCε/nPKCε ratio significantly increases after stimulation resulting in contraction in cPKCβI inhibitor peptide βIV5-3 preincubated muscles. Data are mean percentage ± SEM, ^*^p < 0.05 (*n* = 5).

We analyzed pMunc18-1 levels in nerve stimulated contracting muscles preincubated with the peptide εV_1−2_. We found a significant decrease in pMunc18-1 levels without changes in Munc18-1 (Figure 9A). In concordance, the ratio of pMunc18-1/Munc18-1 significantly decreased (−20.59 ± 5.10; *p* < 0.05). Thus, nPKCε could phosphorylate Munc18-1 during contraction. On the other hand, we analyzed the action of cPKCβI. βIV_5−3_ peptide decreased pMunc18-1, increased Munc18-1 and the ratio pMunc18-1/Munc18-1 decreased (−48.55 ± 10.10; *p* < 0.05) (Figure 9B). This result indicates that during contraction, cPKCβI could contribute to increase Munc18-1 phosphorylation and downregulate its synthesis. Also, it is possible that Munc18-1 accumulates when cPKCβI is blocked because of the fall in its phosphorylation under muscle contraction.

Figure 9C shows that βIV5-3 peptide significantly enhances pnPKCε and decreases nPKCε during contraction. These data indicate that cPKCβI inhibits nPKCε activity, which results in a significantly increased ratio pnPKCε/nPKCε (102.96 ± 9.65; *p* < 0.05).

Altogether, nerve stimulation increases pMunc18-1 and muscle contraction prevents the effect of stimulation on Munc18-1 and pMunc18-1. Moreover, nPKCε phosphorylates Munc18-1 both in basal and synaptic activity conditions (with and without contraction), being higher the effect without contraction. The cPKCβI inhibits nPKCε phosphorylation activity in all the previous conditions. Interestingly, during muscle contraction, the role of the cPKCβI shifts to upregulate pMunc18-1.

**Figure 10:**
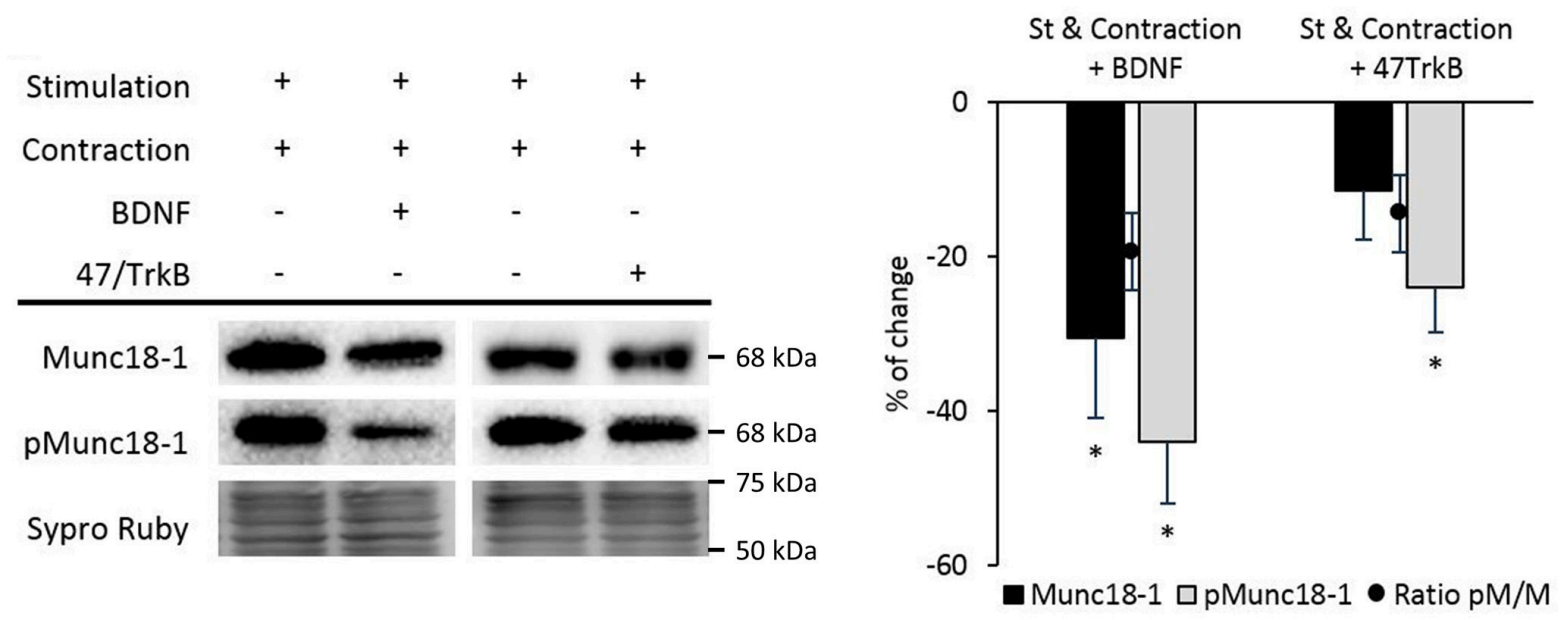
BDNF/TrkB signaling pathway modulates Munc18-1 phosphorylation under muscle contraction conditions. Representative Western blot bands and quantification show that both exogenous BDNF and 47/TrkB significantly decrease pMunc18-1 levels after stimulation resulting in contraction. Moreover, exogenous BDNF decreases Munc18-1 levels under these conditions. Data are mean percentage ± SEM, ^*^p < 0.05 (*n* = 5).

We described above that the continued stimulation and contraction activity caused an important fall of pMunc18-1 after 30 min. During contraction, 47/TrkB did not affect Munc18-1 but increased pMunc18-1. This result suggests that pMunc18-1 depends on TrkB signaling. Surprisingly, exogenous BDNF during contraction significantly decreased pMunc18-1 and Munc18-1 protein levels (Figure 10). Thus, these results demonstrate that muscle contraction prevents Munc18-1 phosphorylation against the activity of nPKCε, cPKCβI, and BDNF/TrkB.

## Discussion

In the neuromuscular system, presynaptic PKCs are fundamental for the neuromuscular function. In particular, synaptic activity couples presynaptic exclusive cPKCβI and nPKCε to acetylcholine release through the BDNF/TrkB pathway (Besalduch et al., 2010; Obis et al., 2015a; Hurtado et al., 2017b). It could be suggested that one or both of these isoforms could regulate Munc18-1 phosphorylation in an activity-dependent way. Thus, in this study, we localized Munc18-1 at the NMJ and we investigated whether cPKCβI and/or nPKCε regulate Munc18-1 phosphorylation. The results demonstrate that phosphorylation of Munc18-1 at the NMJ is increased in response to a signaling mechanism initiated with synaptic activity and directly mediated by nPKCε while cPKCβI and TrkB activities work to prevent this synaptic activity–induced Munc18-1 phosphorylation. On the other side, muscle contraction prevents Munc18-1 phosphorylation when a continuous stimulation is performed.

### Munc18-1 and pMunc18-1 in the adult skeletal muscle: expression, location and regulation by calcium and PMA

#### Munc18-1 presence in the nerve terminal of the NMJ

Munc18-1 in mammals is mainly expressed in neurons and neuroendocrine cells (Hata et al., 1993; Garcia et al., 1994; Pevsner et al., 1994b; de Vries et al., 2000) while other isoforms display a scattered distribution outside the nervous tissue (Hata and Südhof, 1995; Katagiri et al., 1995; Tellam et al., 1995; Halachmi and Lev, 1996; Riento et al., 1996; Yu et al., 2014). We immunolabeled muscles to colocalize Munc18-1 with NMJ markers (syntaxin for the axon and presynaptic terminal, S-100 for the Schwann cell, and AChR for the postsynaptic membrane in the myocyte). Munc18-1 is localized at the presynaptic element, above the postsynaptic gutters and colocalizes with syntaxin, confirming its presence in the nerve terminal. Moreover, there is higher immunosignal in concrete areas, which may correspond to places close to the active zones, where syntaxin realizes its function with the help of Munc18-1 (Garcia et al., 1994; Pevsner et al., 1994a; Dulubova et al., 1999; Yang et al., 2000). Furthermore, the absence of Munc18-1 in the Schwann cell nor the muscle tissue confirms the neural location in the diaphragm.

#### Calcium and PMA promote Munc18-1 phosphorylation

The PKC phosphorylation of Munc18-1 Ser-306 and Ser-313 was determined *in vitro* to reduce the ability of Munc18-1 to bind to syntaxin (Fujita et al., 1996) and in intact cells to change the kinetics of vesicle fusion and release (de Vries et al., 2000; Barclay et al., 2003). In contrast with muscle, we found modest pMunc18-1 levels in the CNS as previously reported in resting neurons and cromaffin cells (de Vries et al., 2000; Barclay et al., 2003; Craig et al., 2003). This could be a specific functional adaptation in muscle tissue related with the high number of synaptic vesicles released per impulse and the high quantal content of the resultant postsynaptic potentials compared with CNS synapses (Wood and Slater, 2001). However, PMA is able to increase pMunc18-1/Munc18-1 ratio in the CNS but not in muscle, which could indicate a specific functional adaptation in this tissue.

The increase of phosphorylated Munc18-1 after PMA treatment proves that PKC regulates Munc18-1 phosphorylation also in the NMJ, as it was described in adrenal chromaffin cells and synaptosomes (Barclay et al., 2003; Craig et al., 2003). It has been demonstrated that the effects of phorbol esters on neurotransmission are due to its binding to Munc13 and via PKC and that this two pathways converge (Rhee et al., 2002; Wierda et al., 2007). We now provide new evidence that Munc18-1, which has a key role in neurotransmission, is also affected by phorbol esters through PKCs. Interestingly, PMA and nerve stimulation (see later) increased Munc18-1 protein levels, which suggests that PKC activation promotes the expression of the protein besides its phosphorylation. This PKC implication in Munc18-1 levels is further discussed below. In contrast, stimulating PKC with 5 mM Ca^2+^, a dose that results in a higher level of transmitter release at the NMJ (Santafé et al., 2001, 2007), increased the ratio pMunc18-1/Munc18-1. This difference could be explained by PMA ability to stimulate all PKC isoforms, resulting in the increase of expression and phosphorylation of Munc18-1, while calcium only enhances the activation of conventional PKC isoforms, such as cPKCα and cPKCβI, which may do not affect directly Munc18-1 expression. The existence of multiple PKC substrates makes it necessary to explain neurotransmission through the interaction between the different components. Munc18-1 is only a part of the mechanism but PKC has other neurotransmission-related substrates, including SNAP-25 (Genoud et al., 1999; Hepp et al., 2002), Synaptotagmin (De Jong et al., 2016), N-ethylmaleimide-sensitive factor (NSF) (Matveeva et al., 2001; Pontier et al., 2006; Chou et al., 2010), N-type calcium channels (Barrett and Rittenhouse, 2000), tetrodotoxin (TTX)-transient and hNav1.7 voltage-gated Na^+^ channels (Curia et al., 2007; Tan et al., 2014) and myristoylated alanine-rich C-kinase substrate (MARCKS) (Obis et al., 2015a). Preliminary unpublished results drive us to think that PMA effects extend to SNAP25 and synaptotagmin in the NMJ.

### Synaptic activity modulates Munc18-1 and its phosphorylation through nPKCε and cPKCβI isoforms and BDNF/TrkB signaling pathway

#### Synaptic activity enhances Munc18-1 and its phosphorylation

Our results show that a moderate increase in nerve activity increases cytosolic Munc18-1 and pMunc18-1 levels in the NMJ. This result coincides with previous ones showing that depolarization of the nerve terminal in synaptosomes triggers Munc18-1 phosphorylation (de Vries et al., 2000). Synaptic activity-induced increase of total levels of Munc18-1 could be explained by an increase of its synthesis or alternatively by a decrease on its degradation. Although stimulation redistributes Munc18-1 and pMunc18-1 to the cytosol (de Vries et al., 2000; Cijsouw et al., 2014), they remain principally in the membrane like in basal conditions. This could be because pMunc18-1, once released from syntaxin, can be linked to other membrane proteins such as Rab3A, Mint1/2 and neurexins (Biederer and Südhof, 2000; Huang et al., 2011). This result supports the hypothesis of the permanent assembly of Munc18-1 with the SNARE complex contributing to organize the docking-fusion sites.

After 30 min of synaptic activity we observed the result of the adaptative steady changes of a very rapid mechanism of vesicle movements. In fact, changes in pMunc18-1 at 1 min of stimulation (60 stimuli) are only revealed in the absence of phosphatase activity. Therefore, during synaptic activity, the phospho-dephosphorylation balance tilts toward accumulating Munc18-1. This indicates that phosphatase activity plays a role at short stimulation times whereas prolongated stimulation activity continuously increases pMunc18-1 level to be finally sustained. This effect may be related with a positive adaptive plasticity.

Altogether, our results show that stimulation increases both total and phosphorylated Munc18-1 in muscles, similarly to the PMA treatment. This suggests that Munc18-1 phosphorylation may be directly modulated by PKC during synaptic activity.

#### nPKCε enhances pMunc18-1 both in basal and synaptic activity conditions

nPKCε and cPKCβI are exclusively located in the nerve terminal of the NMJ, regulated by synaptic activity and involved in neurotransmitter release (Besalduch et al., 2010; Obis et al., 2015a,b; Hurtado et al., 2017b). Here we found that these two isoforms are involved in the phosphorylation of Munc18-1. Our results demonstrate that nPKCε enhances Munc18-1 phosphorylation in basal conditions and under stimulation (with and without contraction, see later). Therefore, the effect of nPKCε over neurotransmission could be produced through Munc18-1 phosphorylation. In accordance with our results, pnPKCε is decreased during activity (Obis et al., 2015a), indicating that the nPKCε-induced increase of pMunc18-1 after synaptic activity is accompanied by a decrease of nPKCε, probably reflecting its downregulation process after activation (Lee et al., 1996; Lu et al., 1998; Kang et al., 2000; Gould and Newton, 2008; Gould et al., 2009).

#### cPKCβI inhibits pMunc18-1 during synaptic activity

We previously reported that pcPKCβI increases in the membrane fraction while total cPKCβI is downregulated during synaptic activity indicating that synaptic activity induces cPKCβI activation (Hurtado et al., 2017a,b). Here we found that synaptic activity-dependent cPKCβI function downregulates pMunc18-1 levels. Interestingly, its activity is also necessary for the increase of pMunc18-1 by nerve stimulation and nPKCε. The results also show that cPKCβI inhibits nPKCε. Thus, we propose that when cPKCβI is blocked, pMunc18-1 values are higher because nPKCε operates without the cPKCβI inhibition.

Furthermore, in basal conditions, cPKCβI increases Munc18-1 levels without affecting its phosphorylation. Interestingly, cPKCβI is not the only factor increasing Munc18-1, as synaptic activity still increases it in the presence of the βIV_5−3_ peptide (Figure 4, last column). Therefore, cPKCβI is not an essential factor for synaptic activity to increase Munc18-1 levels.

The nPKCε inhibitor peptide in basal conditions reduces Munc18-1 levels. Because Munc18-1 is confined in the nerve terminal of the NMJ (Figure 2) it can only be increased by enhancing translation. The inhibitor peptides disrupt PKC-RACK1 interaction, a complex which regulates translation acting directly to the ribosome (Larburu et al., 2016). Particularly, in the CNS, PKCε/RACK1 promotes mRNA stability and translation (Alkon et al., 2005; Hongpaisan et al., 2013) and PKCβII/RACK1 promotes ribosome activation through eIF6 phosphorylation (Ceci et al., 2003). On the other hand, nPKCε inhibitor peptide during stimulation increases total Munc18-1 levels. The action of pMunc18-1 during stimulation induces its degradation (Schmitz et al., 2016). Consequently, when Munc18-1 is no longer phosphorylated, it accumulates due to lack of functionality (Schmitz et al., 2016). Our results suggest that this mechanism compensates the translation effect observed in basal conditions.

In summary, our results indicate that nPKCε upregulates and cPKCβI downregulates Munc18-1 phosphorylation during synaptic activity at the NMJ. Therefore, a balance between the interdependent activities of both isoforms can be a relevant cue to regulate the exocytotic apparatus.

#### BDNF/TrkB pathway downregulates pMunc18-1 during synaptic activity

BDNF is produced after muscular contraction both *in vitro* (Matthews et al., 2009) and *in vivo* (Hurtado et al., 2017b). In the NMJ, synaptic activity enhances BDNF expression, as basal activity is required to maintain its levels (Gómez-Pinilla et al., 2002). Also, synaptic activity enhances the BDNF/TrkB signaling pathway to increase cPKCβI activity which is related with acetylcholine release (Hurtado et al., 2017b). Moreover, exogenous BDNF influences synaptic plasticity (Schinder, 2000; Aguado, 2003). Altogether, BDNF/TrkB signaling is a key regulator of neuromuscular activity (Gómez-Pinilla et al., 2002; Mantilla et al., 2004; Garcia et al., 2010).

During synaptic activity, endogenous BDNF downregulates Munc18-1 phosphorylation through TrkB. However, exogenous BDNF cannot affect Munc18-1 phosphorylation. Because of that, we propose that TrkB receptor could be more accessible for endogenous BDNF than for exogenous. Also, TrkB pathway could be saturated by endogenous BDNF production, preventing additional effects of exogenous BDNF. Interestingly, exogenous BDNF decreases also pMunc18-1 levels in basal conditions (results not shown). Thus, in basal conditions, there is low endogenous BDNF production and TrkB receptors are free to receive exogenous BDNF. This downregulates pMunc18-1 as synaptic activity-increased endogenous BDNF does. This is in accordance with the fact that exogenous BDNF did not change cPKCβI and nPKCε phosphorylation and activation in stimulated muscles (Obis et al., 2015a; Hurtado et al., 2017b).

As stated, synaptic activity enhances the BDNF/TrkB pathway to increase cPKCβI activity (Hurtado et al., 2017b). On the contrary, TrkB activity decreases phosphorylation of nPKCε and pMunc18-1. Therefore, it seems that TrkB enhances pcPKCβI activity to prevent nPKCε-mediated Munc18-1 phosphorylation.

### Muscle contraction prevents pMunc18-1 against the activity of nPKCε, cPKCβI, and BDNF/TrkB

Increasing evidence indicate that muscle contraction has a retrograde effect on the NMJ probably through BDNF (Matthews et al., 2009; Hurtado et al., 2017b). It has been suggested that a retrograde factor from the myasthenic postsynapse might stimulate presynaptic Munc18-1 activity (Sons et al., 2003). Here we found that physiological synaptic activity (nerve stimulation with the resulting contraction) contributes to modulate pMunc18-1 levels in a different way than without contraction. Therefore, some adaptive changes mediated by contraction decrease pMunc18-1 levels, suggesting that there is a retrograde factor that negatively controls presynaptic Munc18-1 and pMunc18-1.

There is a complex change in nPKCε, cPKCβI, and Munc18-1 interactions when synaptic activity ends up in muscle contraction. It seems that cPKCβI shifts to increase pMunc18-1 after 30 min of contraction, which is opposite to what is observed in stimulation without contraction. Muscle contraction induced similar effects on TrkB and cPKCβI than presynaptic activity, suggesting that both molecules work coordinately. Therefore, there is a similar shift in the coupling of the endogenous BDNF/TrkB signaling and cPKCβI to promote Munc18-1 phosphorylation. Thus, TrkB pathway enhances Munc18-1 phosphorylation during contraction like cPKCβI and nPKCε. Altogether, it seems that the muscle contraction-induced retrograde factor that prevents the synaptic activity-induced increase of pMunc18-1 is TrkB independent.

Is thought-provoking that muscle contraction reduces Munc18-1 and pMunc18-1 but, in the same condition, cPKCβI, nPKCε, and TrkB increase pMunc18-1. Surprisingly, exogenous BDNF decreases pMunc18-1 and Munc18-1 during contraction, which indicates that exogenous and endogenous BDNF act differently. Probably, the exogenous is binding a different receptor, such as p75^NTR^. The result also reinforces the idea that it has the same effect as contraction. BDNF effect could act through the same pathway that muscle contraction triggers or another one. Evidence support that muscle contraction induces BDNF synthesis (Matthews et al., 2009; Hurtado et al., 2017b). In this regard, the present results indicate that the pathway triggered by muscle contraction is not saturated at the moderate frequency of stimulation of 1 Hz and can be enhanced adding exogenous BDNF. Although the interpretation of the data is complex, we observed that in the experiments blocking TrkB, the total Munc18-1 did not depend of this receptor. Thus, the shift of pcPKCβI on Munc18-1 phosphorylation between synaptic activity and muscle contraction could be the key element that allows the decrease of pMunc18-1 after contraction, which could be considered as an adaptation. This effect seems to occur in parallel with the nPKCε, cPKCβI, and the endogenous BDNF-TrkB pathway positive effects over Munc18-1 phosphorylation.

**Figure 11:**
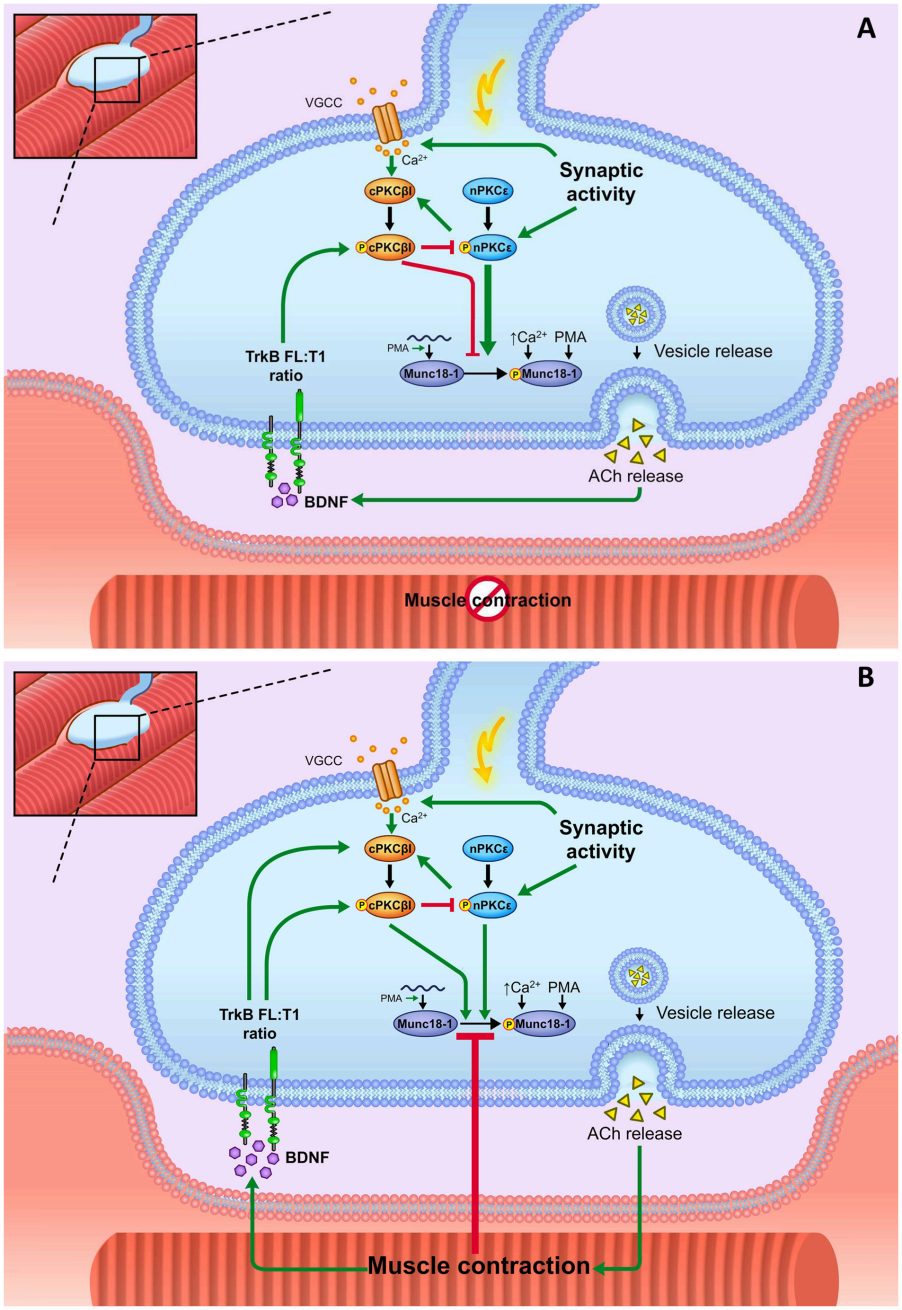
Summary. Munc18-1 is expressed and phosphorylated in basal conditions in the skeletal muscle. It is placed in the motor nerve terminals and absent in the Schwann cells and muscle cells. Munc18-1 phosphorylation at the residue Ser-313 occurs in response to extracellular Ca^2+^ increase and to PKC activation, either pharmacologic (PMA) or physiologically induced. Presynaptic stimulation induces calcium influx, which promotes the activation of cPKCβI. This isoform downregulates the nPKCε phosphorylation whereas nPKCε isoform enhances cPKCβI. Both nPKCε and cPKCβI isoforms contribute to regulate Munc18-1 phosphorylation during synaptic activity. **(A)** Representation of how BDNF/TrkB signaling, nPKCε, and cPKCβI modulate Munc18-1 under presynaptic stimulation without contraction. In stimulation protocols, synaptic activity results in pnPKCε enhancing Munc18-1 phosphorylation and cPKCβI decreasing it, maybe through negatively regulating the action of pnPKCε. Thus, the balance between the activities of these two isoforms can be a relevant cue in the regulation of the exocytotic apparatus. pnPKCε activity and pMunc18-1 level are positively related and negatively modulated by the TrkB receptor. TrkB would enhance pcPKCβI activity to prevent the synaptic activity-induced Munc18-1 phosphorylation mediated by nPKCε. **(B)** Representation of how BDNF/TrkB signaling, nPKCε and cPKCβI modulate Munc18-1 under presynaptic stimulation with contraction. Muscle contraction prevents the synaptic activity–induced Munc18-1 phosphorylation through a mechanism that decreases the nPKCε/cPKCβI/TrkB signaling.

Figure 11 shows a diagram that summarizes the results of this study. In brief, nerve stimulation increases phosphorylation of Munc18-1 on Ser-313 and the resulting nerve-induced muscle contraction prevents the effect of nerve stimulation by itself on Munc18-1 and pMunc18-1 levels. Moreover, nPKCε positively regulates Munc18-1 phosphorylation both in basal conditions and in synaptic activity conditions (with and without contraction) being higher the effect of nPKCε in synaptic activity without contraction. The cPKCβI isoform negatively regulates pnPKCε phosphorylation activity in all the studied activity conditions. However, during contraction, the role of the cPKCβI shifts to increase pMunc18-1. BDNF/TrkB signaling has a similar effect than cPKCβI in all the activity conditions studied, suggesting the coordinated regulation of both pathways.

## Concluding remarks

Munc18-1 is a synaptic exocytotic molecule, phosphorylated by PKC, essential for appropriate neurotransmitter secretion, whose regulation by PKCs and synaptic activity is little known *in vivo*. Here we demonstrate that the synaptic activity-dependent phosphorylation of Munc18-1 at the NMJ is regulated by the BDNF/TrkB signaling through nPKCε and cPKCβI isoforms and that muscle contraction prevents it. These results provide a molecular insight about how neuromuscular activity influences neurotrophic control to phosphorylate Munc18-1 and how this retrograde signaling regulates specific presynaptic PKC isoforms.

## Author contributions

AS data collection, quantitative analysis, literature search, data interpretation, statistics. VC-M literature search, data interpretation, design graphic abstract. LJ-B literature search, data interpretation. LN, EH, and MT data interpretation. JT, ML, and NG conception and design, literature search, data interpretation, manuscript preparation.

### Conflict of interest statement

The authors declare that the research was conducted in the absence of any commercial or financial relationships that could be construed as a potential conflict of interest.

## Abbreviations

Ach: Acetylcholine
AChRs: acetylcholine receptors
AR: Adenosine receptors
BDNF: brain-derived neurotrophic factor
CNS: central nervous system
cPKCβI: conventional protein kinase C beta I
βIV5-3: cPKCβI-specific translocation inhibitor peptide
EPPs: evoked endplate potentials
GAPDH: glyceraldehyde-3-phosphate dehydrogenase
HRP: horseradish peroxidase
LAL: levator auris longus
Munc18: mammalian uncoordinated-18
mAChR: muscarinic acetylcholine receptors
NSF: N-ethylmaleimide-sensitive factor
NMJ: neuromuscular junction
NT-4: neurotrophin-4
nPKCε: novel protein kinase C epsilon
εV1-2: nPKCε-specific translocation inhibitor peptide
p75 ^NTR^: p75 neurotrophin receptor
PBS: phosphate buffer saline
PKA: protein kinase A
PKC: protein kinase C
PMA: phorbol 12-myristate 13-acetate
PVDF: polyvinylidene difluoride
RACK: receptor for activated C-kinase
TRITC: tetramethylrhodamine
TrkB: tropomyosin receptor kinase B
α-BTX: α-bungarotoxin
μ-CgTx-GIIIB: μ-conotoxin GIIIB
VDCC: voltage-dependent calcium channels.
